# First Early Hominin from Central Africa (Ishango, Democratic Republic of Congo)

**DOI:** 10.1371/journal.pone.0084652

**Published:** 2014-01-10

**Authors:** Isabelle Crevecoeur, Matthew M. Skinner, Shara E. Bailey, Philipp Gunz, Silvia Bortoluzzi, Alison S. Brooks, Christian Burlet, Els Cornelissen, Nora De Clerck, Bruno Maureille, Patrick Semal, Yves Vanbrabant, Bernard Wood

**Affiliations:** 1 Unité Mixte de Recherche 5199, de la Préhistoire à l’Actuel: Culture, Environnement et Anthropologie (UMR 5199 - PACEA), Centre National de la Recherche Scientifique (CNRS), Université de Bordeaux, Talence, France; 2 Department of Anthropology, University College London, London, United Kingdom; 3 Center for the Study of Human Origins, Department of Anthropology, New York University, New York, New York, United States of America; 4 Max Planck Institute for Evolutionary Anthropology, Department of Human Evolution, Leipzig, Germany; 5 Laboratori di Antropologia, Dipartimento di Biologia Evoluzionistica “Leo Pardi”, Università di Firenze, Florence, Italy; 6 Center for the Advanced Study of Hominid Paleobiology, Department of Anthropology, The George Washington University, Washington DC, United States of America; 7 Geological Survey of Belgium, Royal Belgian Institute for Natural Sciences, Brussels, Belgium; 8 Section of Prehistory and Archaeology, Royal Museum for Central Africa, Tervuren, Belgium; 9 Microtomography CT Research Group, University of Antwerp, Wilrijk, Belgium; 10 Anthropology and Prehistory, Royal Belgian Institute for Natural Sciences, Brussels, Belgium; University of Delaware, United States of America

## Abstract

Despite uncontested evidence for fossils belonging to the early hominin genus *Australopithecus* in East Africa from at least 4.2 million years ago (Ma), and from Chad by 3.5 Ma, thus far there has been no convincing evidence of *Australopithecus*, *Paranthropus* or early *Homo* from the western (Albertine) branch of the Rift Valley. Here we report the discovery of an isolated upper molar (#Ish25) from the Western Rift Valley site of Ishango in Central Africa in a derived context, overlying beds dated to between ca. 2.6 to 2.0 Ma. We used µCT imaging to compare its external and internal macro-morphology to upper molars of australopiths, and fossil and recent *Homo*. We show that the size and shape of the enamel-dentine junction (EDJ) surface discriminate between Plio-Pleistocene and post-Lower Pleistocene hominins, and that the Ishango molar clusters with australopiths and early *Homo* from East and southern Africa. A reassessment of the archaeological context of the specimen is consistent with the morphological evidence and suggest that early hominins were occupying this region by at least 2 Ma.

## Introduction

**Figure 1:**
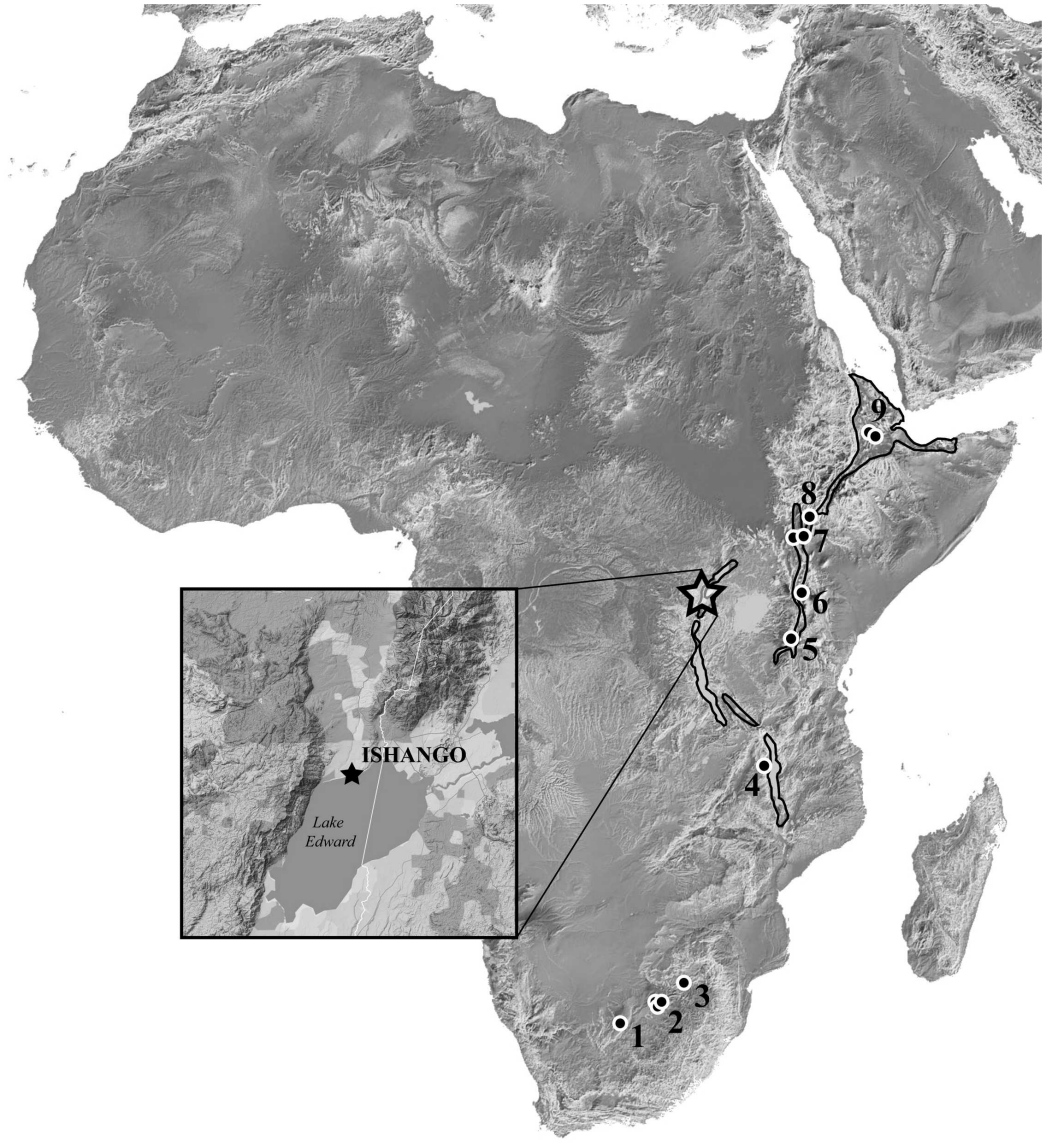
Geographic locations of Ishango and other African localities of early hominins fossils from the Plio-Pleistocene transition period, ca. 2.6–1.8 Ma (*i.e. Au. africanus, Au. garhi, Au. sediba, H. habilis, H. rudolfensis, P. aethiopicus, P. boisei, P. robustus*). Outlined in bold are the western and eastern branches of the African Rift. 1: Taung (South Africa); 2: Drimolen, Gladysvale, Gondolin, Kromdraai, Sterkfontein & Swartkrans (South Africa); 3: Malapa (South Africa); 4: Malema, Uraha (Malawi); 5: Olduvai (Tanzania); 6: Chemeron (Kenya); 7: West and East Turkana, Koobi Fora (Kenya); 8: Omo (Ethiopia); 9: Bouri, Hadar (Ethiopia).

The discovery of *Australopithecus bahrelghazali* [1] in Chad was significant because it extended the known range of this genus to the west of the East African Rift, where the earliest australopiths are documented to at least 4.2 Ma ago [2]. Thereafter, for most of the Plio-Pleistocene, fossil evidence of at least one species of hominin, and at times several hominin species, is found at sites in East and southern Africa [3]–[6] (Figure 1). There has been extensive debate about the role played by environmental factors in the regional and temporal distributions of early hominin taxa [7]–[11], but as yet, the Western (Albertine) Rift Valley, which today lies on the boundary between the tropical rain forest of the Congo basin and the savannas and woodlands of East Africa [12]–[14] has played little role in these debates. The Albertine Rift experienced several climatic changes at approximately 3 Ma, 2.6 Ma and 1.8 Ma that led to the partial replacement of flora and fauna of Congolian affinities with flora and fauna with similarities to East Africa that are adapted to more open conditions [13]. During some of this period simple core and flake artifacts possibly associated with faunas that date to 2.4–2.0 Ma on biostratigraphic grounds suggest the presence of Plio-Pleistocene hominins in the Western Rift Valley of the Democratic Republic of Congo (DRC) ([15],Text S1 in File S1). Evidence from Ugandan part of the Western Rift Valley may also indicate Lower Pleistocene occupation of this region [13]. However, the stratigraphic context of the finds from the Semliki Valley (*i.e.* Kanyatsi, Senga 5A) has been questioned ([16], Text S1 in File S1), and apart from a cemented block of cranial fragments and a worn molar in Western Uganda of Lower or possibly early Middle Pleistocene age provisionally attributed to *Homo cf. erectus* [13], [17], no pre-Upper Pleistocene hominin fossil evidence is known from this region.

This paper describes an unworn first upper molar (M^1^) (#Ish25) that the authors believe is the first compelling evidence of Plio-Pleistocene hominins in the Western Rift Valley in Central Africa. Macro and micro scale analyses have demonstrated the relevance of dental morphology for hominin taxonomy and phylogeny reconstruction (*e.g.* [18]–[23]). However, the ongoing debate about the taxonomy of the earliest members of the genus *Homo* - whether or not they should be removed from the clade [24]–[25] - and the growing complexity of Plio-Pleistocene hominin taxonomic diversity [26]–[27], outline the challenge of identifying isolated dental remains to a specific taxon. Here we use µ-CT imaging to compare the external and internal macro-morphology of this M^1^ to those of a large sample of australopiths, and fossil and recent *Homo*. Our results suggest that this tooth, which most closely resembles Plio-Pleistocene hominins, provides new insights about the presence of early hominins in Central Africa.

### Ishango Site

The #Ish25 left M^1^ was found at Ishango 11 (IS-11) an archaeological site in the Semliki valley located in the DRC part of the Western Rift. This valley is best known for its Late Stone Age (LSA) artifacts and particularly for the early evidence for harpoons [28], but the recovery of stone tools *in situ* in the Semliki Formation (Late Lower to Middle Pleistocene) at Katanda 2, and possibly also in the Plio-Pleistocene Lusso Formation (first known as Kaiso beds [29]) at Kanyatsi 2 and Senga 5A, suggested that early hominins were present in the valley long before the Upper Pleistocene ([15], Text S1 in File S1).

**Figure 2:**
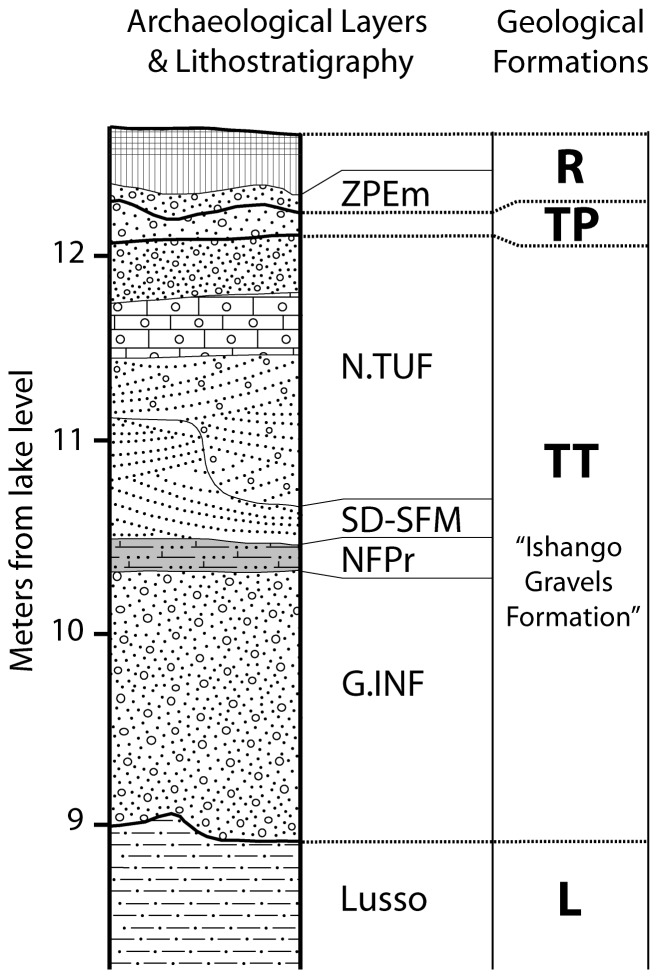
Stratigraphic section of Ishango 11 at the junction of de Heinzelin’s trenches (modified from [31]). See Text S1.2 and Figure S2 (in File S1) for the detailed description of the lithology. ZPEm = Post-Emersion Zone; N.TUF = Tuffaceous Levels; SD-SFM = Hardened Sand - Fine Micaceous Sand, NFPr = Principal Fossiliferous Level, G.INF = Inferior Gravels, Lusso = Lusso Beds, R = Recent, TP = Museya Gravels Formations, TT = Ishango Gravels Formation, L = Lusso Formation.

Ishango 11 is located along the Semliki River where it flows out of Lake Rutanzige (or Lake Edward; see [12] p.3). The site was initially recognized and superficially explored in 1935 by Damas [30], but the first formal excavations were carried out by de Heinzelin in the 1950s during his geological and archaeological expeditions in the Upper Semliki region [30]–[33]. At Ishango, de Heinzelin excavated two perpendicular trenches as well as the area to the west of their junction (Text S2.1 and Figure S1 in File S1); the stratigraphy he established was later confirmed by the work of the Semliki Research Expedition [34]–[35]. Recent to Upper Pleistocene lithostratigraphic units compose a terrace complex TT (or *Terrasse tuffacée*) later redefined as the Ishango Gravels Formation [16] that truncated and overlies the Plio-Pleistocene Lusso Formation (Figure 2, Text S2.2 and Figure S2 in File S1). The latter have been dated using biochronology to between 2.6 Ma and 2.0 Ma ([13]–[14], [36]–[42], Text S2.2 in File S1). Towards the base of the Ishango Gravels Formation, overlying the ca. 1m thick basal gravels (G.INF), a 10–20 cm thick *Niveau Fossilifère Principal* (NFPr) yielded an early Late Stone Age (LSA) assemblage consisting of numerous lithic and bone artifacts as well as faunal remains ([31], [35], [43]–[49], Text S2.2 in File S1) in association with more than a hundred heavily mineralized hominin remains. Radiometric dating of the LSA layer (NFPr) yielded an age between 25–19 Ka ([34]–[35], [50], Text S2.2 in File S1), an age consistent with that suggested by characteristics of the faunal assemblage [48]. During the initial study of the 1950s LSA hominin remains, it was assumed that all were attributed to *Homo sapiens*. However, the exceptionally large size of a left M^1^ (#Ish25) described by Twiesselmann [43], raised the possibility that evidence of a more primitive hominin may have been mixed in with a fossil assemblage that is otherwise similar to anatomically modern humans.

## Results and Discussion

### Origin of the Tooth

Although the precise location of the archeological samples and the description of their content were recorded by de Heinzelin in his notebook, there was no mention of an isolated tooth. Archival research, however, has revealed that #Ish25 was discovered the first day of de Heinzelin’s excavation, and that it came from the corner area, west of the junction of the trenches (Text S2.3 and Figures S3–S8 in File S1). This area preserved only the basal parts of the Upper Pleistocene Ishango Gravels Formation, namely levels G.INF and NFPr ([31], Text S2.3 in File S1). In the archives of the excavation, de Heinzelin indicated that intrusive fossils from the Lusso Formation had been reworked into the lower layers of the Ishango Gravels Formation (Figure S7 in File S1: “G.INF = Inferior gravels with few fossils, rare harpoons and reworked Kaiso fossils”). In 1955 and 1957, de Heinzelin described the G.INF layer as an unstratified mass of rolled gravels including numerous fragments of reworked fossils from the Lusso/Kaiso Formation (brown bullhead, crocodile, etc.) mixed together with fauna and artefacts accounting for the first stages of the Ishango civilization ([31]–[32], Text S2.3 and Figures S2 and S7 in File S1). The same observations were made by Greenwood [44] and Hopwood & Misonne [45] for the fish and mammal assemblage at the base of the Ishango Gravels Formation. The analysis of the fauna by Peters confirmed the heterogeneity of the basal layers from the Ishango Gravels Formation [48]. He identified reworked intrusive elements (shell fragments) in the G.INF and the NFPr deposits that originate from the older sediments. Since the presence of reworked fossils from the Lusso Formation is confirmed by archaeozoological studies in both basal layers of the Ishango Gravels Formation, this is a legitimate reason to argue that #Ish25 likely derives from the underlying Plio-Pleistocene Lusso Formation and should not be grouped with the LSA hominin fossils.

### Raman Spectroscopy

In order to test the hypothesis that #Ish25 may be a reworked element within the Ishango Gravels Formation, we used Raman spectroscopy, a non-destructive technique, to analyze and compare the diagenetic processes at work in this tooth with those seen in the three teeth from the LSA level that best represented the range of taphonomic alterations seen in fossils from this horizon (Text S3 and Figure S9 in File S1).

**Figure 3:**
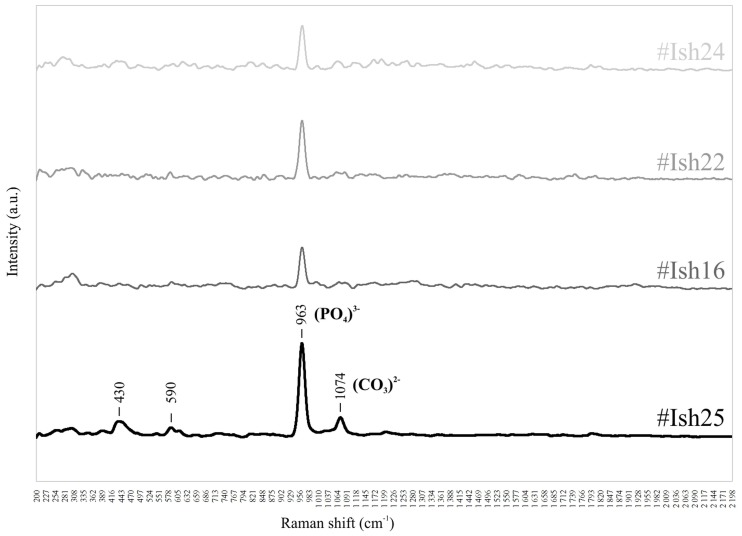
Average Raman spectra of three teeth from the NFPr layer, and of #Ish25.

The Raman spectra acquired from the NFPr material show signatures consistent with dentine spectra ([51]–[52], Figure 3). The three teeth show a similar diagenetic signal, with a 963.5 cm^−1^ peak (υ_1_(PO_4_)^3−^) with a mean signal-to-noise ratio (S/N) of 1250 (ranging from 967 to 1368); there is no evidence of (PO_4_)^3−^ secondary features. The spectra from #Ish25 show an intense 963 cm^−1^ peak (mean S/N of 2152) and a 1074 cm^−1^ peak (υ_1_ (CO_3_)^2−^) with a mean S/N of 456; (PO_4_)^3−^ secondary features at 430 cm^−1^ and 590 cm^−1^ (respectively υ_2_(PO_4_)^3−^ and υ_4_(PO_4_)^3−^) are also present (Text S3 in File S1). The results of the Raman spectroscopy suggest that #Ish25 has a distinctive Raman spectrum compared to the three teeth from the NFPr layer. In particular, #Ish25 has a doubled intensity for the (υ_1_(PO_4_)^3−^) phosphate peak and a clear carbonate signal, suggesting a difference in hydroxyapatite re-crystallization and carbonate integration. This supports the hypothesis that #Ish25 has a different diagenetic history than the remains from the NFPr layer, and is consistent with it being an intrusive element within the basal section of the Upper Pleistocene terrace.

The results from the Raman spectroscopy, plus the archival and geological evidence (Texts S1–S3 in File S1), are consistent with #Ish25 being a reworked element from the Lusso Formation.

### Comparative Morphometric Analyses of Ishango First Upper Molar

**Figure 4:**
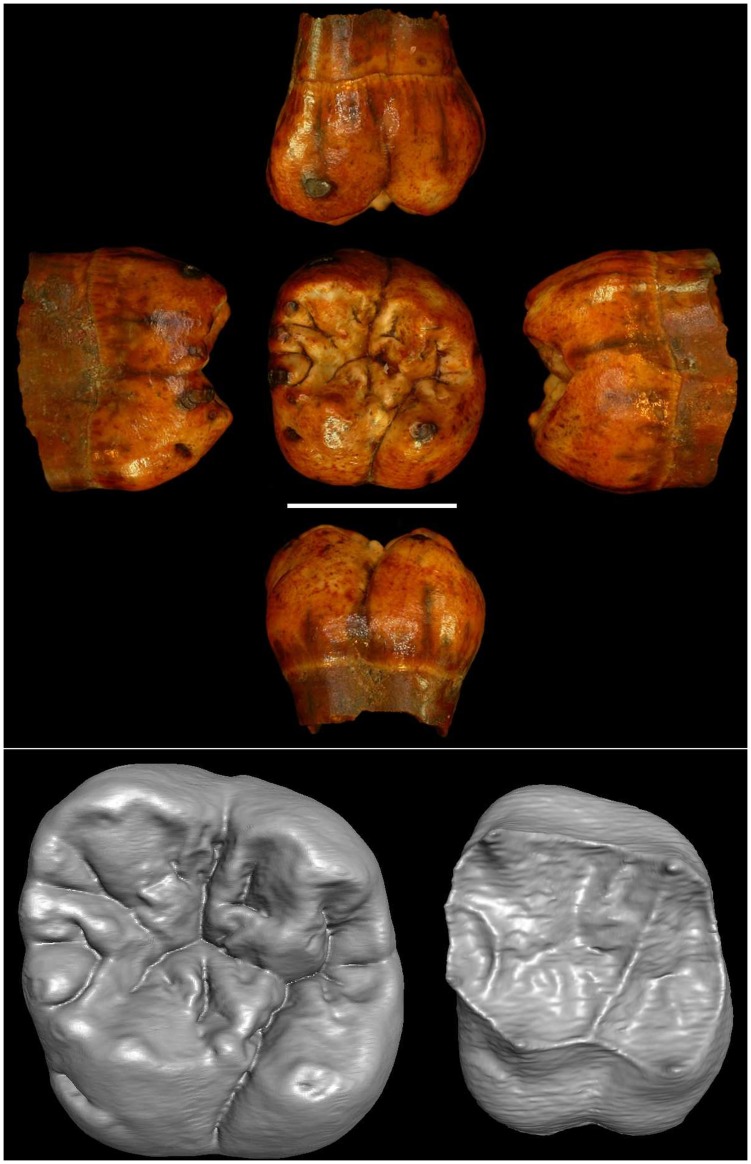
Photograph and three-dimensional reconstruction of Ishango left upper first molar (#Ish25). Upper part, from top to bottom: buccal, mesial, occlusal, lateral and lingual views. Scale bar = 1 cm. Lower part, three-dimensional model of the outer enamel surface (left) and the enamel-dentine junction (right).

The morphological characteristics of #Ish25 molar at the outer enamel surface (OES) and at the enamel-dentine junction (EDJ) confirm its primitive status ([18], Figure 4).

The stage of root formation (scale E; [53]), together with the absence of interproximal wear facets, suggests that it belongs to an immature individual and that the tooth was not fully erupted. The shape of the distobuccal corner of the crown, the relative equivalence of the mesiolingual-distobuccal and mesiobuccal-distolingual axes, the slightly larger dimension of the lingual face compared to the buccal one, and the triradiate fissure pattern in the central fossa of the occlusal surface indicate that this tooth is a first upper molar [54]–[55]. The identification of #Ish25 as a first molar is also supported by analysis of EDJ shape (see below and Text S4.4 and Tables S5–S7 in File S1).

The outer enamel surface of #Ish25 is marked by two crests - a C-shaped anterior transverse crest that joins the mesial marginal ridge (MMR) and a notched, but uninterrupted, *crista obliqua* between the protocone and the metacone. A third crest, a trigonal-hypocone crest that results in a shallow groove at this location on the OES, is only visible on the EDJ surface. The mesial marginal ridge bears three tubercles at the OES that correspond with dentine horn-like projections at the EDJ [56]. There is no metaconule (or cusp 5). A furrow-like Carabelli structure is visible on the lingual OES of #Ish25 with a corresponding depression at the EDJ.

The taxonomic implications of non-metric enamel and dentine characters have been debated [18], [20], [54]. The tubercles on the MMR have limited significance [56], and the expressions of the three crests on #Ish25 have been interpreted as primitive features [18]. The continuous *crista obliqua* is observed in a minority of recent modern humans at the OES (ca. 20%); it is most frequent in early *Homo* and it is also developed in hyper-megadont archaic hominins [18], [20]. The particular C-shaped anterior transverse crest on the EDJ is found in less than 2% of modern human individuals, and it is also rare among australopiths [18]. While the incidence of a furrow-like Carabelli structure in modern human populations varies ca. 12% to ca. 44% [57], its occurrence in early hominins is higher among early *Homo* (ca. 33%) than *Australopithecus* and *Paranthropus* (ca. 18.5%) [58].

The exceptional dimensions of the #Ish25 crown have been noted since its discovery [43], [59]. The mesiodistal and buccolingual diameters of #Ish25 align it with australopiths rather than with Pleistocene *Homo* and recent modern humans (Text S4.1, Table S1 and Figure S10 in File S1). The crown base area of the #Ish25 falls at the upper end of the early *Homo* variation and between the means of *Australopithecus* and *Paranthropus* (Text S4.2 and Table S2 in File S1). The cusp areas of #Ish25 have the following relative size relationships: protocone >paracone>metacone>hypocone. The ratio between the size of the paracone and the metacone separates *Australopithecus* and early *Homo* from later Pleistocene *Homo* [60]. In #Ish25 the paracone is 5.5% larger than the metacone, a relationship that aligns this individual with later *Homo* specimens (Figures S11–S12 in File S1). However, the complexity of the mesial marginal ridge morphology complicates this assessment, for it is not clear whether the diagnostic paracone/metacone relationship holds true for teeth with as many accessory cusps/cuspules as are observed in #Ish25.

Since the relative proportions of enamel and dentine have been used to assess hominid phylogeny, taxonomy and adaptation (*e.g.* [19], [61]–[64]), we further investigated the two- and three-dimensional dental tissue proportions of #Ish25 through micro-computed tomography. In Tables S3 and S4 (in File S1) we compare the results with the available data on hominin upper molars. With the exception of the Neanderthals ([61], Text S4.3 in File S1), dental tissue proportions similar to modern humans are documented back to the Middle Pleistocene [65]–[66], whereas australopiths are characterized by thicker enamel [67]–[68]. The proportions of enamel and dentine exhibited by #Ish25 are closer to the pattern seen in early hominins than to the values seen in both Middle-to-Upper Pleistocene *Homo* and recent humans. In relation to its crown size, the enamel thickness of #Ish25 is comparable to that of the M^1^s from Sterkfontein (*i.e.* Sts 57) and Swartkrans (*i.e.* SK 832) (Figures S13–S14 in File S1).

**Figure 5:**
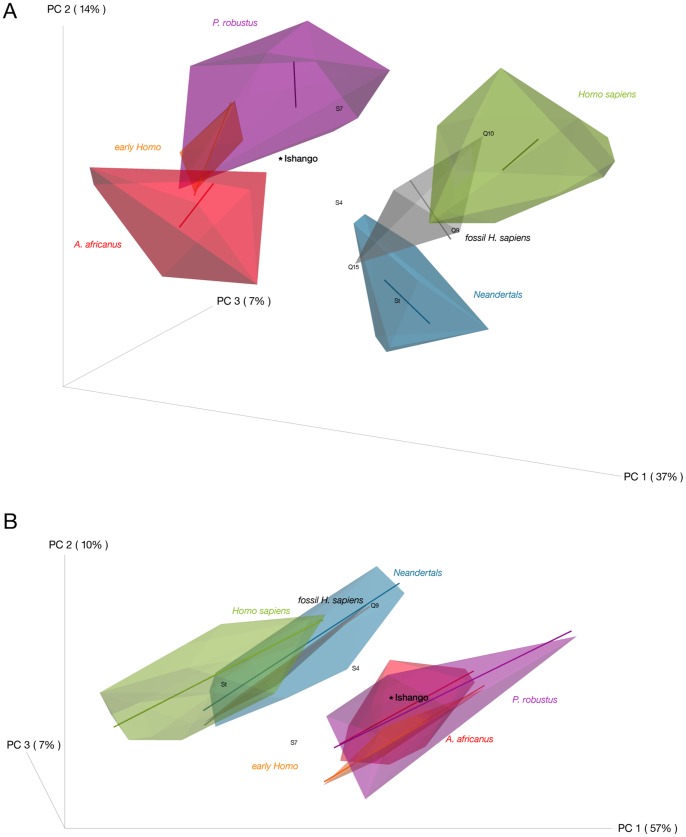
Results of the principal component analysis of first molar EDJ shape in both shape (A) and form (B) space. Projection of the first three principal components of the PCA of the enamel-dentine junction (EDJ) morphology. Solid lines in each convex hull represent static allometric trajectories for respective groups. Abbreviations: Sangiran specimens (S4, S7), Qafzeh specimens (Q9, Q10, Q15) and Steinheim (St).

We used geometric morphometrics to examine the shape of the #Ish25 EDJ based on landmarks and semilandmarks ([23], [69], Text S4.4 and Figure S15 in File S1). A principal component analysis of the Procrustes coordinates in both shape and form space (*i.e.* including also tooth size; see Text S4.4 in File S1) shows a clear separation on the first axis between the Pliocene-Lower Pleistocene hominins and the Middle Pleistocene-recent specimens (Figure 5), with #Ish25 clustering with the former group at the interface of the *P. robustus* and *A. africanus* convex hulls. A cross-validated canonical variates analysis of EDJ shape classifies #Ish25 as most similar in morphology to the early *Homo* comparative sample (Text S4.4 in File S1), while a nearest neighbor analysis links #Ish25 with the *A. africanus* specimen Sts 8 (not illustrated). A comparison of the EDJ shape of the #Ish25 with the mean shape of the post-Lower Pleistocene sample and the mean shape of the Plio-Pleistocene sample indicates that the relative size and position of the dentine horns of the four main cusps (the ridge curve) and the shape of the cervix of #Ish25 match more closely the mean of the Plio-Pleistocene sample. This pattern is even more pronounced in form space, which includes tooth size in the comparison (Figure S16 in File S1). While it is clear that the EDJ shape of the Ishango molar is consistent with it belonging to an early hominin taxon, the lack of a more comprehensive early *Homo* EDJ sample prevents definitive assignment to a specific taxon.

### Conclusions

A reassessment of the Ishango archaeological collections has highlighted the uniqueness of #Ish25, a particularly large hominin first upper molar. The combination of archival evidence about its geological context and the results of Raman spectroscopic analysis suggest that #Ish25 does not belong to the Upper Pleistocene LSA modern human assemblage. With the exception of the derived relative cusp proportions, the external and internal dental morphology of #Ish25 resembles that of australopiths or early *Homo* and the absolute and relative dimensions of the crown and its relatively thick enamel align it with East and southern African early hominins. Finally, in a detailed analysis of the EDJ, which discriminates between australopiths/early *Homo* and post-Lower Pleistocene *Homo*, #Ish25 clusters with the former.

The attribution of #Ish25 to an early hominin has several implications. Locally, this occurrence is consistent with archaeological evidence suggesting that early hominins were in the Semliki valley close to, if not prior to, two million years before the present. More globally, our understanding of early hominin evolution, adaptation and dispersion during the Plio-Pleistocene period is mainly based on fossil evidence from just two regions within the vast African continent, the Eastern Rift Valley from Ethiopia to Tanzania, and southern Africa. The #Ish25 first upper molar is meager, but compelling, evidence that by ca. 2 Ma early hominins had expanded their geographical range into the Western Rift valley of Central Africa, and had occupied a region whose environment has been reconstructed as a woodland to grassland ecotone adjacent to dense lowland forests [14], [70].

Overall, the evidence from Ishango provides a new perspective on hominin morphological and ecological diversity during the Plio-Pleistocene and contributes to our understanding of the patterns of dispersal and evolution of early hominins.

## Materials and Methods

The upper molar #Ish25 belongs to the Ishango collection (inventory number IG 22295) housed in the department of Anthropology and Prehistory in the Royal Belgian Institute of Natural Sciences (RBINS), Brussels, Belgium.

We are grateful to the following institutions and persons that gave permission to study the comparative material. In the following cases, the institution was the legal repository for the fossil material: Archéologie andennaise, Belgium (D. Bonjean), Senckenberg Research Institute (F. Schrenk and O. Kullmer), Croatian Museum of Natural History (J. Radovčić), Ditsong National Museum of Natural History (S. Potze), Institut de Paléontologie Humaine (H. de Lumley, D. Grimaud-Hervé), Institutul de Antropologie “Francisc I. Rainer” (A. D. Soficaru), Max Planck Institute for Evolutionary Anthropology (J.-J. Hublin), Museo Nacional de Ciencias Naturales (A. Rosas), Musée d’Angoulême (J.-F. Tournepiche), Musée d’Archéologie Nationale, National Museums of Kenya (E. Mbua), Musée National de Préhistoire (J.-J. Cleyet-Merle), Rockefeller Museum, Sackler School of Medicine (Y. Rak, A. Barash, I. Hershkovitz), University of Witwatersrand (B. Zipfel), Staatliches Museum für Naturkunde (R. Ziegler), Rheinisches Landesmuseum (H. Joachim), Russian Academy of Science Archaeology Institute (T. Balueva), National Museum of Archaeology in Lisbon, Iziko South African Museum. In the case of the British Museum (N. Spencer, D. Antoine), the Department of Anthropology in the Colorado University in Boulder (D. Van Gerven), and the Royal Belgian Institute of Natural Sciences, the specimen(s) was/were donated to the institution. The specimens from the Department of Anthropology of the National Museum of Natural History (Smithsonian Institution, D. Hunt) are on loan. S. Prat and H. Roche gave access to the specimen in their care. Finally, the comparative material from the Museum für Vor- und Frühgeschichte, Staatliche Museum zu Berlin (A. Hoffmann & W. Menghin) was purchased by this institution.

We used a 785 nm (NIR) laser Raman spectrometer (Senterra, Olympus BX51, Bruker optics) for the analysis of the Ishango teeth. The spectra were acquired using a 2 mW 785 nm laser, during 3×10 s and with a 50×1000 µm spectrometer slit. Raman spectra fluorescence removal and curve-fitting techniques were applied to each of the acquired spectra to overcome the fluorescence problem when Raman spectroscopy is applied to human remains [71]. Confocal Raman microscopy, in a slightly out of focus position, reduced the influence of the resin coating. Several spectra (∼10) were collected on the dentine surface of each tooth and the average used for comparison.

The micro-computed tomography (µ-CT) of #Ish25 was performed at the Scan Research Group Laboratory at the University of Antwerp, Belgium. The specimen was scanned with the SkyScan 1173 high energy spiral X-ray microtomograph with a tube voltage of 130 kV (61 µA current and a projection each 0.2° of rotation) and a resolution of 10 µm. In order to facilitate processing, the volume was re-sampled to a voxel size of 40 µm.

The µ-CT data set of #Ish25 can be downloaded at a resolution of 20 µm following this URL: http://africanarchaeology.naturalsciences.be/archaeological-sites/dem.-rep.-congo-zaire/Ishango/IV.%20Collections/2.%20Human%20Remains/c.%20Files%203D/teeth/ishango-25.

Threshold values between segmented tissues were determined following the half-maximum height methods [72]–[73] using Aviso 6.1 (www.vsg3d.com). The two-dimensional dental tissue proportions of #Ish25 were taken on the virtual mesial cross-section of the tooth following the method developed by Martin [74] and using ArteCore (©2004–2006 ART+COM AG). Three-dimensional data were recorded following the protocols defined by Kono [64], Tafforeau [75] and Olejniczak [76] using Amira 5 (©2008 Visage Imaging, Inc.). The cervical plane (to measure coronal dentine and coronal pulp) was computed following the definitions of Olejniczak [62], [76].

Regarding the comparative sample, the taxonomic attributions of *Australopithecus* and early *Homo* M^1^ follow Wood & Engelman [20], Wood [77], Quam *et al.* [60] and Clarke [78]. Comparative assessment of #Ish25’s external crown dimensions and proportions was performed using classical linear measurements [79] and the method described by Quam *et al.* [60]. The comparative data were compiled from published data and original fossils (Texts S4.1 and S4.2 in File S1). The mean and range of variation of the comparative groups are given in Table S1 (in File S1) and Table S2 (in File S1). Two-dimensional dental tissue proportion data from #Ish25 were compared to published data on M^1^s from australopiths, and fossil and recent *Homo* [64]–[65], [67]–[68], [74], [80] in Table S3 (in File S1) and Figure S13 (in File S1). The recent modern human sample (RMH) was compiled by mathematically combining the sub-sample means and standard deviations using the formula of Cleuvenot & Houët [81]. The upper molar comparative samples used in the 3D dental tissue proportions analyses come from Olejniczak *et al.* [61]–[62], [68]. Although several studies have emphasized comparisons of dental tissue proportions between teeth of similar positions [63], [82], we used combined samples of upper molars to maximize the comparative sample size (Table S4 in File S1). With respect to the geometric morphometric analysis, the process by which landmarks and semilandmarks [83]–[84] was generated and compared for each specimen is detailed in the Text S4.4 (in File S1). Landmarks were placed around the cervix of the crown and around the ridge curves that link the dentine horns of the protocone, paracone, metacone and hypocone. The comparative sample, which includes Pleistocene and recent *Homo sapiens*, Neandertals, *Homo erectus* from Indonesia, early *Homo* from east and southern Africa, *Paranthropus robustus*, and *Australopithecus africanus* is given in Table S5 (in File S1).

## Supporting Information

File S1 **File includes supporting text, supporting figures, and supporting tables.**
supporting text:
Text S1. Previous evidence of hominin occupation in the Western Rift Valley
Text S2. Ishango Site
Text S2.1 History of excavations
Text S2.2 Stratigraphy and Dating
Text S2.3 Origin of #Ish25 upper molar
Text S3. Raman spectroscopic analysis
Text S4. Morphometric comparison
Text S4.1 Crown dimensions
Text S4.2 Cusp area analysis
Text S4.3 Two- and three-dimensional dental tissue proportions
Text S4.4 Enamel-dentine junction (EDJ) morphology References
Supporting Figures:
Figure S1. The excavation plan at Ishango 11
Figure S2. Schematized stratigraphic section of the ten first meters of Ishango N43GE trench
Figure S3. Letter from de Heinzelin to Twiesselmann dated to Sunday 23rd of April 1950. Recto
Figure S4. Letter from de Heinzelin to Twiesselmann dated to Sunday 23rd of April 1950. Verso
Figure S5. First page of the Heinzelin notebook
Figure S6. Letter from de Heinzelin to the director of the RBINS dated to the 16th of February 1951
Figure S7. First sketch of a stratigraphical section with the definition of the archaeological layers
Figure S8. First map of the Ishango excavation dated from the 5th to 9th of May 1950
Figure S9. Photographs of the teeth from the LSA human assemblage (NFPr level) that were used as comparative samples for the Raman spectroscopic analyses
Figure S10. Bivariate plot of the crown diameters of #Ish25 and the comparative sample (*cf.* Table S1)
Figure S11. Bivariate plot of the relative paracone area in relation to the relative metacone area. Comparative samples as in Table S2
Figure S12. Scatter plot of the first and second principal components of the PCA on relative cusp areas. Comparative samples as in Table S2
Figure S13. Adjusted Z-scores of the two-dimensional dental tissue proportions of #Ish25
Figure S14. Bivariate plot of the volume of coronal dentine (DPVOL) and the relative enamel thickness (RET3D)
Figure S15. Illustration of landmarks collected on the ridge curve, cervix curve and main dentine horns
Figure S16. Comparison of the EDJ shape of #Ish25
Supporting Tables:
Table S1. Crown dimensions (mm) of #Ish25 and the comparative group means and standard deviations
Table S2. Comparison of crown and cusp areas between Ishango #Ish25 and comparative fossil groups (mm^2^)
Table S3. Two-dimensional dental tissue proportions of M^1^ #Ish25 and the first upper molar comparative samples
Table S4. Three-dimensional dental tissue proportion of M^1^ #Ish25 and the pooled upper molar comparative samples
Table S5. First molar sample used to analyze EDJ shape of the #Ish25 molar
Table S6. Second molar sample used to assess the classification of #Ish25 as a first molar
Table S7. Classification of the M1/M2 comparative sample using a cross-validated CVA
(DOC)
