# Supplementary material for: First Early Hominin from Central Africa (Ishango, Democratic Republic of Congo)
Source: PLoS One. 2014 Jan 10;9(1):e84652. doi: 10.1371/journal.pone.0084652 (PMC3888414; doi:10.1371/journal.pone.0084652)
Supplement: File S1 — File includes supporting text, supporting figures, and supporting tables. supporting text: Text S1. Previous evidence of hominin occupation in the Western Rift Valley Text S2. Ishango Site Text S2.1 History of excavations Text S2.2 Stratigraphy and Dating Text S2.3 Origin of #Ish25 upper molar Text S3. Raman spectroscopic analysis Text S4. Morphometric comparison Text S4.1 Crown dimensions Text S4.2 Cusp area analysis Text S4.3 Two- and three-dimensional dental tissue proportions Text S4.4 Enamel-dentine junction (EDJ) morphology References Supporting Figures: Figure S1. The excavation plan at Ishango 11 Figure S2. Schematized stratigraphic section of the ten first meters of Ishango N43GE trench Figure S3. Letter from de Heinzelin to Twiesselmann dated to Sunday 23rd of April 1950. Recto Figure S4. Letter from de Heinzelin to Twiesselmann dated to Sunday 23rd of April 1950. Verso Figure S5. First page of the Heinzelin notebook Figure S6. Letter from de Heinzelin to the director of the RBINS dated to the 16th of February 1951 Figure S7. First sketch of a stratigraphical section with the definition of the archaeological layers Figure S8. First map of the Ishango excavation dated from the 5th to 9th of May 1950 Figure S9. Photographs of the teeth from the LSA human assemblage (NFPr level) that were used as comparative samples for the Raman spectroscopic analyses Figure S10. Bivariate plot of the crown diameters of #Ish25 and the comparative sample (cf. Table S1) Figure S11. Bivariate plot of the relative paracone area in relation to the relative metacone area. Comparative samples as in Table S2 Figure S12. Scatter plot of the first and second principal components of the PCA on relative cusp areas. Comparative samples as in Table S2 Figure S13. Adjusted Z-scores of the two-dimensional dental tissue proportions of #Ish25 Figure S14. Bivariate plot of the volume of coronal dentine (DPVOL) and the relative enamel thickness (RET3D) Figure S15. Illustration of landmarks coll [file pone.0084652.s001.doc]

**Text S1. Previous evidence of hominin occupation in the Western Rift Valley**

Two relatively well-studied regions of the Western Rift Valley (the Upper Semliki and the Ugandan side of Lake Albert) have yielded long geological sequences with rich fauna and some archaeological remains.

In the Upper Semliki Valley of the Democratic Republic of Congo, the sequence includes a Plio-Pleistocene fluvial lacustrine series (Lusso Formation), a Lower to Middle Pleistocene fluviatile series (Semliki Formation) followed by the Middle to Upper Pleistocene initiation and development of terrace formation along the South flowing Semliki (Kasaka Formation, Katanda Formation, Ishango Gravels Formation, Museya Gravels Formation) [1]. Biface industries with spheroids and some evidence of Levallois technology are documented in several localities within the Semliki Formation [2] or on the subsequent Upper Terrace (Kasaka Formation) of the river. At Katanda, 6 km north of Ishango, in two locations, excavation of an Oldowan assemblage, associated with *Elephas recki* in the upper part of the Semliki Formation indicate that the Semliki Formation in large part does not postdate ca. 500 ka, and is likely much older toward the base [3]. These finds and abundant evidence of artefacts in the Ugandan sites of the Albertine Rift Valley [4] indicate consistent occupation of the Western Rift, presumably by *Homo erectus*, during the late Lower to early Middle Pleistocene.

At Nyabusosi on the Ugandan side of Lake Albert, towards the top of the Behaga Member of the Nyabusosi Formation, cranial fragments and a worn upper molar, cemented into a ferricrete block and attributed provisionally to “*Homo cf. erectus*” were recovered in 1986 by Pickford and Senut [5]. The remains were first dated to 0.7 to 1.8 Ma with a preference for the older age [6], and later revised to "ca. 1.5 Ma" [4] on the basis of the presence of *Kolpochoerus majus* and *Elephas recki* in the fauna collected near the fossil.

The 2.85 - 0.4 Ma time-range of “classic” *E. recki* [7] as well as early Middle Pleistocene dates for both *Elephas recki* and especially *Kolpochoerus majus*, at other east African sites [8-11] suggest that this hominin specimen could also derive from horizons around or even younger than the Lower/Middle Pleistocene boundary at 780 ka.

However, a tuff in the uppermost Kagusa Member, which overlies the Behaga Member of the Nyabusosi Formation, was potentially correlated chemically with a tuff in the Turkana Basin Natoo Member of the Nachikui Formation (1.65 - 1.3 Ma) implying an older age [12]. Therefore some questions remain concerning the age of this hominin specimen.

While hominin presence during the time span of *Homo erectus* is uncontroversial, earlier Plio-Pleistocene hominin occupation is more debated. Within the Semliki Valley, lithic artefacts provisionally associated with the Lusso Formation were described from two locations: Kanyatsi 2 on the lake shore ca. 3 km east of Ishango, and Senga 5A, ca. 10 km north of Ishango along the Semliki River. At Kanyasti, a very small number of core artefacts were recovered either *in situ* or with adhering matrix suggesting attribution to the Lusso Formation. These could also be derived from younger deposits through slumping, although the 1988 excavations did not support this hypothesis [13].

The Senga 5A site is located on a low ca. 11 m terrace comparable to the Ishango terrace, ca. 10 km north of Ishango [13-15]. The entire site exhibits indications that it was formed or at least affected by Upper Pleistocene and Holocene sedimentary processes. However, a large collection (>5000) of diagnostic Plio-Pleistocene mammalian and fish fossils (*e.g. Tragelaphus nakuae, Notochoerus euilus* and *Metridiochoerus jacksonii)* suggesting contemporaneity with the Omo Valley Shungura Formation F-G in the Eastern Rift (2.0 - 2.35 Ma) were recovered from a derived ironstone rubble. These were associated with a much smaller number of artifacts, some of which exhibit typical Lusso Formation iron-staining in the cracks, suggesting possible contemporaneity with the Plio-Pleistocene fossil assemblage [15]. Sediments in the site contained the heavy mineral perovskite, which is a marker of Upper Pleistocene volcanic activity in the nearby Katwe volcanic field. The western portion of the site contained a mixture of Lusso-age fossils and more sub-fossilized specimens of probable Holocene age. On the other hand, faunas from the eastern portion were overwhelmingly of Lusso age and contained little evidence of mixing with extant species. As a whole, the Senga 5A site suggests but does not confirm the possible presence of tool-making hominins in the Semliki by 2 Ma.

**Text S2. Ishango site**

Text S2.1 History of excavations

The site was first identified by Damas during his 1935-1936 expedition in the Upper Semliki, which focused on recent lake fauna and flora [16]. Damas collected some bone material at Ishango that included a fragment of human mandible at a level equivalent to 180-200 cm below the surface in a partially cemented matrix with large mammal bones [17].

In 1950, a new expedition was set up by de Heinzelin in order to find more archaeological sites and human fossils in the Upper Semliki region [2, 18-19]. At Ishango, he excavated two trenches, one parallel (N143GE) and the other perpendicular (N43GE) to the river shoreline (Figure S1). The southeastern edge of the N143GE trench bordered the 1935 survey of Damas [18]. The two trenches met up northwestward at the border of the lake cliff. To the west of their junction, limited by gully erosions, an area of 100 m2 preserved only the lower parts of the archaeological levels (Ishango Gravels Formation, TT). De Heinzelin started his excavation on the 23th of April 1950 in this western zone (Corner W), noting later [18] that most of the human remains, fauna and artefacts came from the area west of the junction between his two trenches (zones A & B on Figure S1). He observed that the archaeological levels gradually disappear all around, though large remnants remain present in both trenches and between the trench N43GE and the cliff.

Further excavations at Ishango include the 1959 extension of the N43GE trench by Splingaer, whose material is unpublished. In 1985-1986, two four-meter-square excavations, one near the junction of the two trenches and one further into the cliff, were carried out by Brooks and members of the Semliki Research Expedition to obtain materials for dating [20-21].


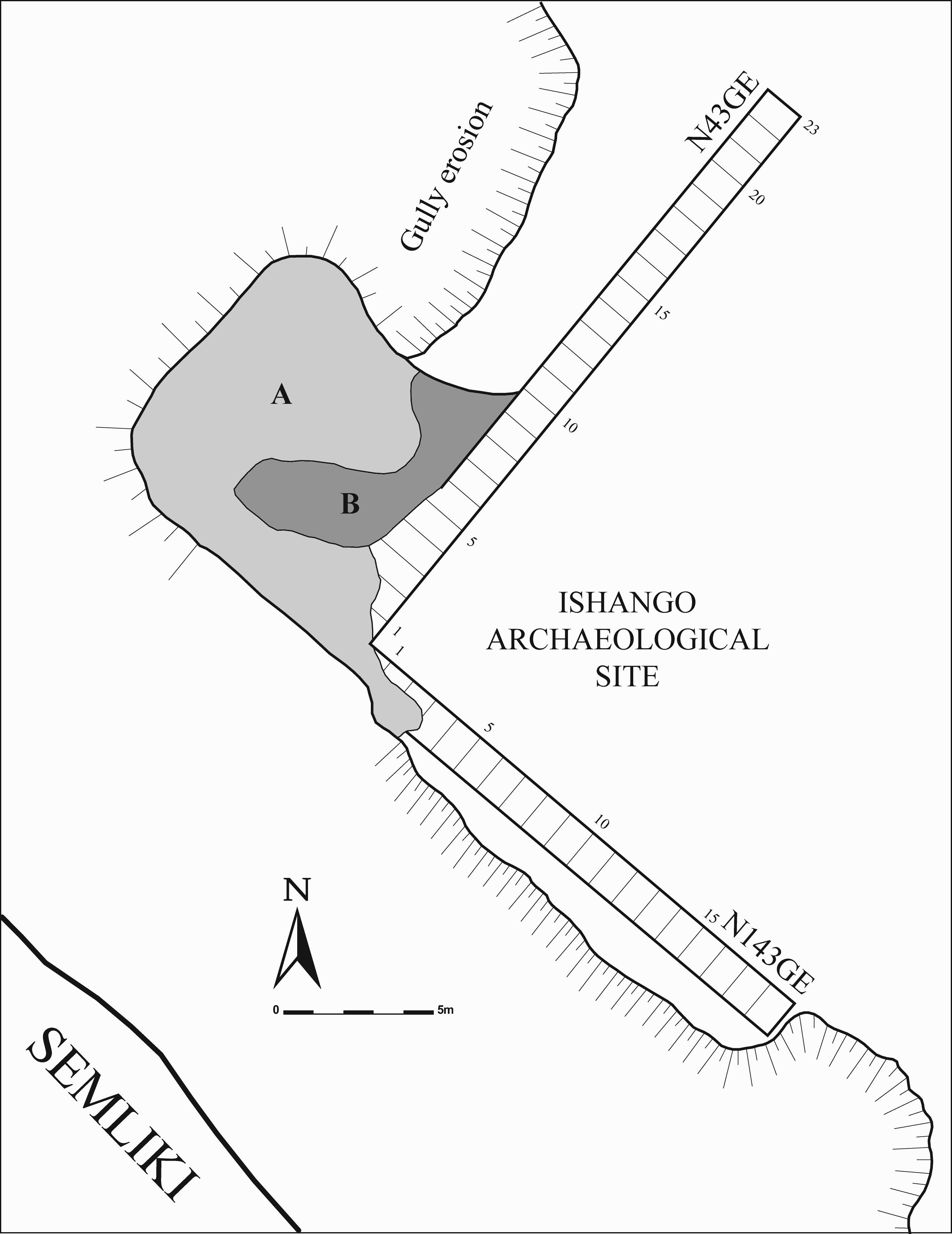


**Figure S1.** The excavation plan at Ishango 11, modified following de Heinzelin [18].

A: area preserving only the basal part of the Ishango Gravels Formation (excavated first, between the 23th of April and the 9th of May 1950. This area was initially named "Field E", then "Field W" and finally "Corner W" in de Heinzelin's notes). B: area where the tuffaceous levels where present in addition to the latter ones (excavated between the 17th and 19th of July 1950). No grid system was laid out in areas A and B.

Text S2.2 Stratigraphy and Dating

The lithostratigraphic units identified at the Ishango 11 (Figure S2) are as follows from top to bottom: R/TP/TT/L [1].

The top part of the sequence are Recent deposits (symbol R) correlated to the uppermost Holocene. At Ishango 11, they include the Post-Emersion Zone (ZPEm) occupation level which was first described by de Heinzelin [19] and dated between 1,680 ± 80 CalBP to 3,170 ± 90 CalBP by ^14^C on charcoals [3].


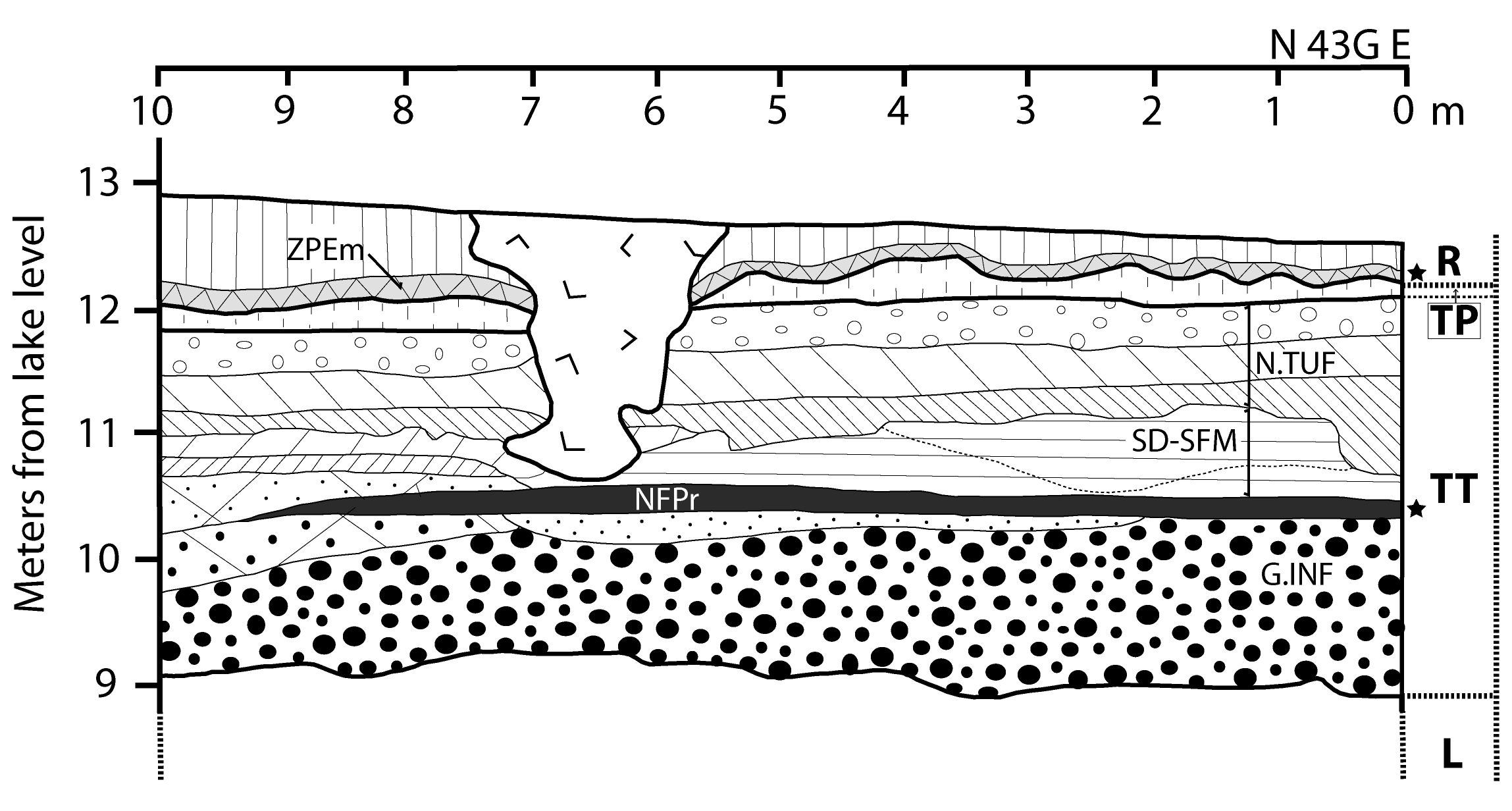
The informal Semliki Lower Terrace Complex (Symbol T) which precedes the Recent deposits regroups the Ishango Gravels Formation (symbol TT) and the Museya Gravels Formation (symbol TP). These fluvial deposits are located at about 9 to 12 m above the present-day Semliki river [1]. The Museya Gravels Formation (symbol TP) is correlated with the middle Holocene and immediately posterior to the volcanic tuffs [1,19]. The stratotype of the Ishango Gravels Formation (symbol TT) has been defined at IS-11 [19]. This fluvial deposit with linked deltaic and slope deposits, and volcanic admixture, is dated to the uppermost Pleistocene to the lower Holocene [1]. At the time of its deposition, the course of the Semliki River was inverted (south-flowing) compared to the present day [18,22].

**Figure S2.** Schematized stratigraphic section of the ten first meters of Ishango N43GE trench, modified following de Heinzelin [1,19].

R: Recent Formation; TP: Museya Gravels Formation; TT: Ishango Gravels Formation; L: Lusso Formation. ZPEm: Post Emersion Zone; N.TUF: Tuffaceous levels; SD-SFM: Hardened Sand - Fine Micaceous Sand; NFPr: Principal Fossiliferous level; G.INF: Inferior Gravels. Stars: archaeological assemblages containing human remains.

The detailed lithological and stratigraphic description of the Ishango Gravels Formation is given by de Heinzelin [18-19]. Four major levels are shown on the trench sections (Figure S2):

- N.TUF (Tuffaceous Levels) refers to a group of layers made of silty fine sand, small white gravels, accumulated shells, all cemented by carbonatation and tuffaceous material. It contains some remains of Late Stone Age assemblages characterized by quartz and quartzite lithic artefacts (*i.e.* microliths, mortars, and grindstones), a bone industry (points and harpoons) and ochre [18-19]. The base of the tuffaceous level has been directly dated by ^14^C on mollusc shell to 20,155 ± 245 CalBP [3].
- SD-SFM (Hardened Sand - Fine Micaceous Sand) corresponds to interstratified, variably cemented, bank of sand. Debris of shell and bones are present with harpoons with one barbed row, some with a few barbs on the opposite side. The contact with the NFPr is variably indurated.
- NFPr (Principal Fossiliferous Level) is the main human occupation layer of the site, consisting of a dense accumulation of bone debris and shells. All the remains are encrusted in their upper part by cement made of fine and saline materials which percolated from the higher layers and precipitated at this level. The bones are heavily mineralized [3]. This level includes abundant early Late Stone Age lithic and bone artefacts, faunal remains (mammal, fish and mollusks) and human remains [18, 21, 23-29]. A series of five radiocarbon dates on ostrich eggshells (OES) and mollusc shell all fall between 25,570 ± 350 CalBP and 19,920 ± 450 CalBP [21, 3, 30]. Analyses of amino acid diagenesis in both the eggshell and mollusk shell samples [21] indicated that these are more appropriate ages than originally thought, especially in the case of ostrich eggshell (alloisoleucine/isoleucine ratio = 0.43 ± 0.04), as OES is unaffected by high levels of non-biogenic carbon in Lake Edward.
- G.INF (Inferior Gravels) are vaguely stratified gravels containing numerous fragments of reworked fossils from the Lusso Formation [19, 24, 27] together with some Later Stone Age artifacts including bone harpoons.

At IS-11, the lower level of the Ishango Gravels Formation (G.INF) cuts the oldest fossiliferous deposits identified in the Upper Semliki basin, the Lusso Formation (symbol L). The Lusso Formation, first identified as Kaiso beds [31], is lake deposit mudstones with sands and ironstones [15-16]. They are rich in mammal, fish and mollusk fossils as well as fossil wood. The faunal assemblages at several sites support an age from the later Pliocene to the earliest Pleistocene. The mollusk assemblage of the Lusso Formation has been correlated to the associations GX and GX' from the Kaiso/Hohwa beds, dated to 2.6 - 2 Ma [4, 32-33]. Some divergences between the Lusso mollusk assemblage and the Ugandan references have been underlined by Pickford *et al.* [4]. They could be related to a slightly earlier age of the Lusso assemblage or to the pene-contemporaneous fragmentation of the Lake Obweruka into two sub-basins (the basins of the proto-lake Edward and of the proto-lake Albert), also suggested by the ichthyofauna [34]. The mammalian fauna indicates a biostratigraphic correlation with the fauna from Members F and G of the Shungura Formation in Ethiopia, between 2.35 and 2 Ma [35-37]. The base of the Lusso Formation has not been observed and the top of the beds is an erosive contact [37].

Text S2.3 Origin of #Ish25 upper molar

De Heinzelin recorded for each geological sample a clear description of contents, which facilitates partial reconstruction of the spatial organization of the human remains. However, no mention of an isolated tooth was found within any of the human samples described in de Heinzelin's notebooks. By cross-checking the information of several archives from the 1950s excavation (Figures S3-S8) the authors were able to establish the origin of #Ish25. Below are selected translations of hand written letters from de Heinzelin to Twiesselmann dated to the very first days of the excavation at Ishango, and the first page of de Heinzelin's notebook:

- Letter to Mister Twiesselmann dated to Sunday 23rd of April 1950 (Figures S3-S4).

Front page - **Black ink**:

*Sunday 23/IV/50*

*Mister Twiesselmann*

*Here ends the first day of excavation. Or rather of clearing. The stratigraphy starts to show up and we already have two harpoons and some worked quartz. the Director must come tomorrow with the headquarters from the Parcs. It's why I have opened the steam from today. Not yet of humans.*

(...)

At the bottom of the front page, under de Heinzelin signature, but with a **Turquoise ink**:

*Good news. I have the base of a human tibia and maybe* ***some teeth****. Results from 1/4 of m3 from the fossiliferous gravel. Tuesday 25th at 10h the Director has not yet arrived.*

Back page - Turquoise ink:

*Report Tuesday night 25:*

*26 harpoons*

*21 fragments of worked quartz unrolled*

*8 fragments of rolled worked quartz*

*Hundreds of broken or worked bone fragments*

*1 complete tibia*

*1 huge base of tibia*

*1 tibia head of a immature individual, detached from the cartilage*

*2 femoral heads very partial human or humanoïd*

*1 humerus fragment Unbelievable mix of human 2 radius fragments and robust traits*

*1 cubitus fragment*

*5 complete or partial foot bones*

*1 parietal or frontal fragment*

*5 portions of cranium probably belonging to a large primate*

*One hundred kg or so of mammal bones, sauria, fishes and maybe birds.*

*I'm crumbling under the weight of the samples and am eaten by the sun, which is a blessing. I suggest that the first cleaning is done on the premises, despite that mobilize workers, otherwise we will never cope in the lab.*

*Not yet any sight of the administrative staff.*

*JdeH.*


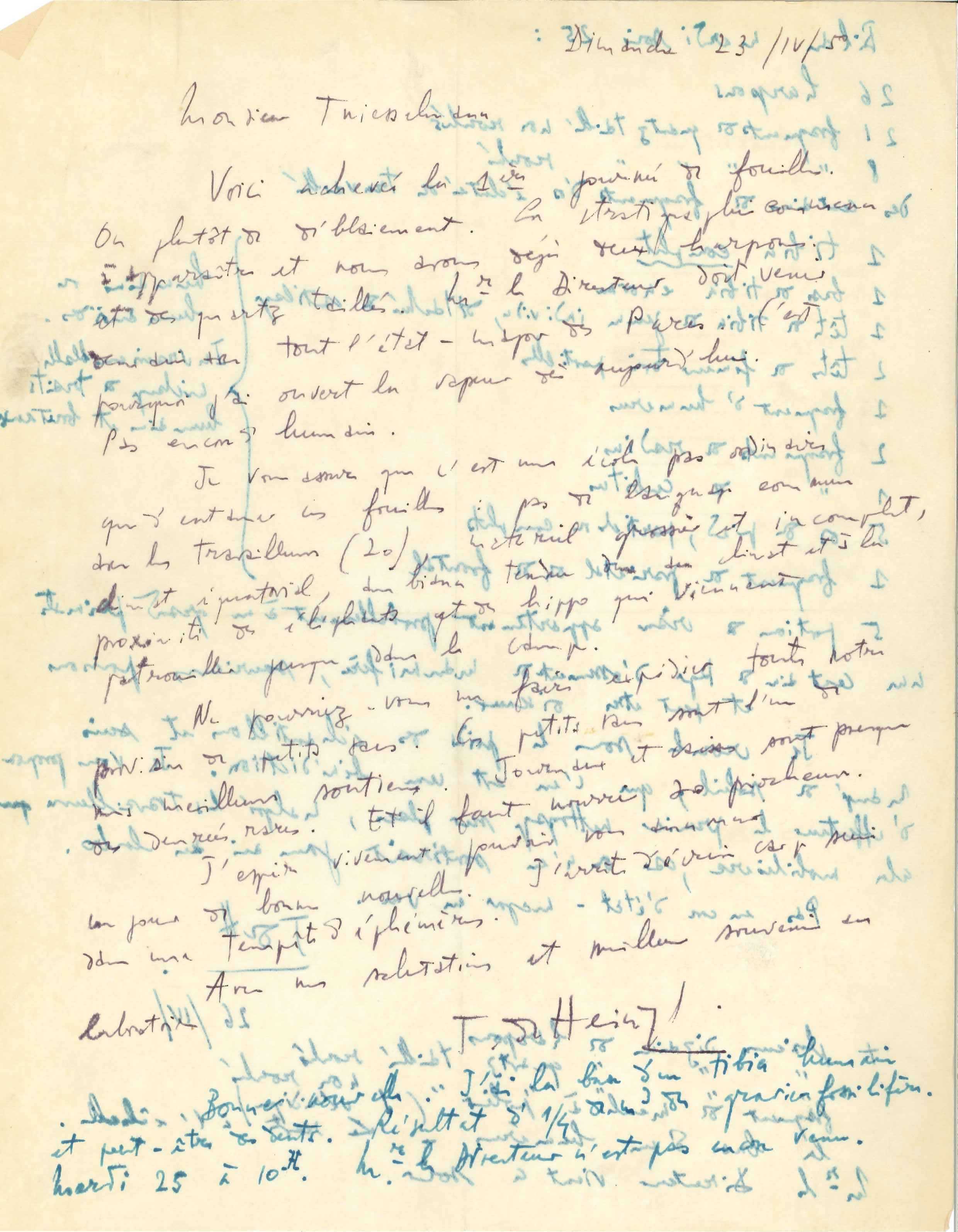


**Figure S3.** Letter from de Heinzelin to Twiesselmann dated to Sunday 23rd of April 1950. Recto.

"*Dimanche 23/IV/50. Monsieur Twiesselmann. Voici achevées les premières journées de fouilles. Ou plutôt de déblaiement. La stratigraphie commence à apparaître et nous avons déjà deux harpons et des quartz taillés. Mr le Directeur doit venir demain avec tout l'état-major des Parcs. C'est pourquoi j'ai ouvert la vapeur dès aujourd'hui. Pas encore d'humain. Je vous assure que c'est une école pas ordinaire que d'entamer ces fouilles : pas de langage commun avec les travailleurs (20), matériel grossier et incomplet, climat équatorial, avec biana(?) tendu du au climat et à la proximité des éléphants et des hippo qui viennent patrouiller jusque dans le camp. Ne pourriez-vous me faire expédier toute notre provision de petits ?acs. Ces petits ? sont l'un de mes meilleurs soutiens. Journaux et caisses sont presque des denrées rares. Et il faut nourrir des piocheurs. J'espère vivement pouvoir vous donner un jour de bonnes nouvelles. J'arrête d'écrire car suis dans une tempête d'éphémères. Avec mes salutations et meilleur souvenir au laboratoire. J. de Heinz.*

*Bonne nouvelle. J'ai la base d'un tibia humain et peut-être* ***des dents****. Résultat d'1/4 de m³ de gravier fossilifère.*

*Mardi 25 à 10 h. Mr le Directeur n'est pas encore venu."*

*
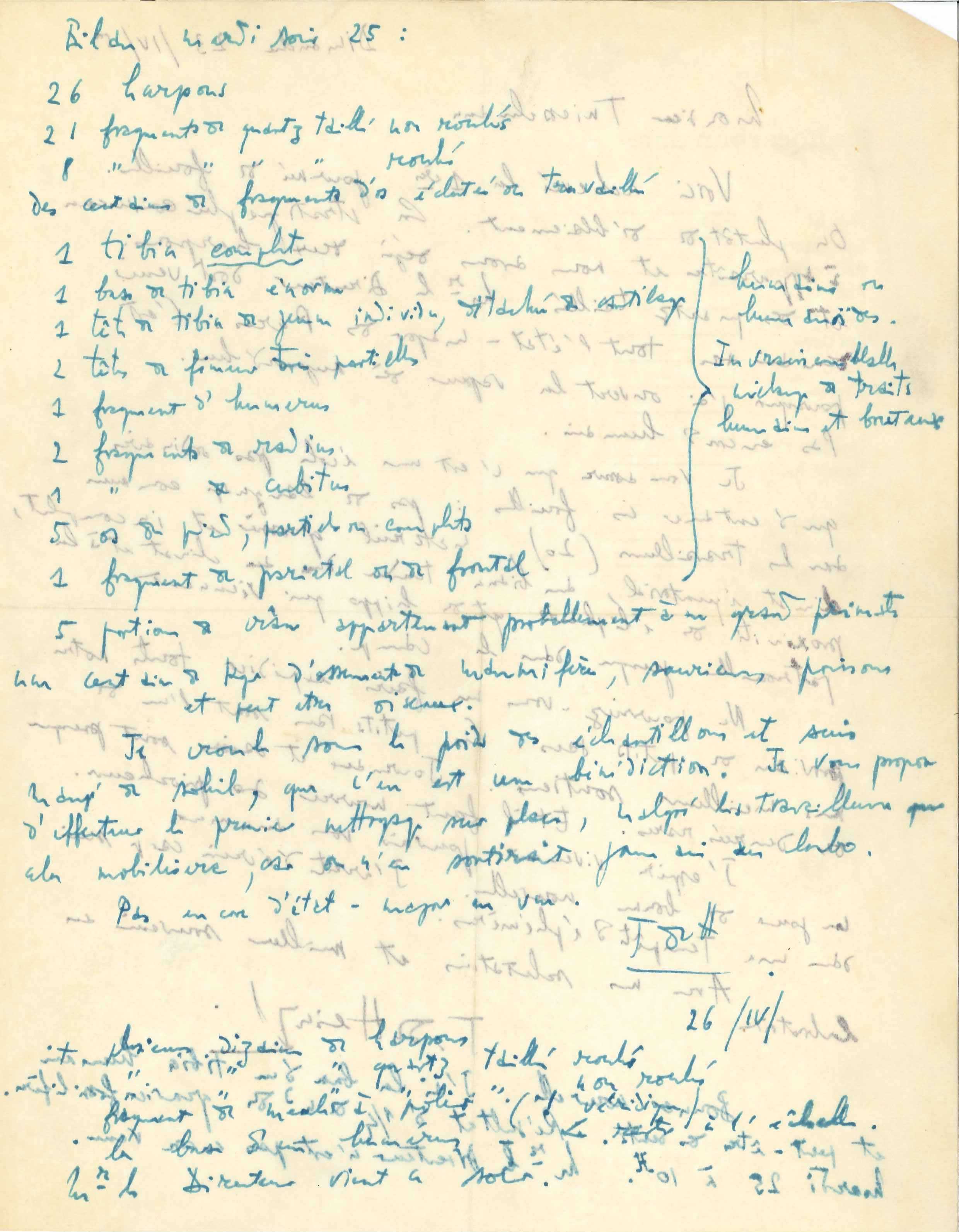
*

**Figure S4.** Letter from de Heinzelin to Twiesselmann dated to Sunday 23rd of April 1950. Verso.

" *Bilan mardi soir 25 :*

*26 harpons*

*21 fragments de quartz taillé non roulés*

*8 '' '' '' '' roulés*

*Des centaines de fragments d'os éclatés ou travaillés*

*1 tibia complet*

*1 base de tibia énorme humains ou*

*1 tête de tibia de jeune individu, détachée du cartilage humanoïdes.*

*2 têtes de fémur très partielles Invraisemblable*

*1 fragment d'humérus mélange de traits*

*2 fragments de radius humains et brutaux*

*1 fragment de cubitus*

*5 os de pied, partiels ou complets*

*1 fragment de pariétal ou de frontal*

*5 portions de crâne appartenant probablement à un grand primate une centaine de kilo d'ossements de mammifères, sauriens, poissons et peut-être oiseaux.*

*Je croule sous le poids des échantillons et suis mangé de soleil, que c'en est une bénédiction. Je vous propose*

*d'effectuer le premier nettoyage sur place, malgré les travailleurs que cela mobilisera, car on n'en sortirait jamais au labo. Pas encore d'état-major en vue. JdeH.(...)"*

*
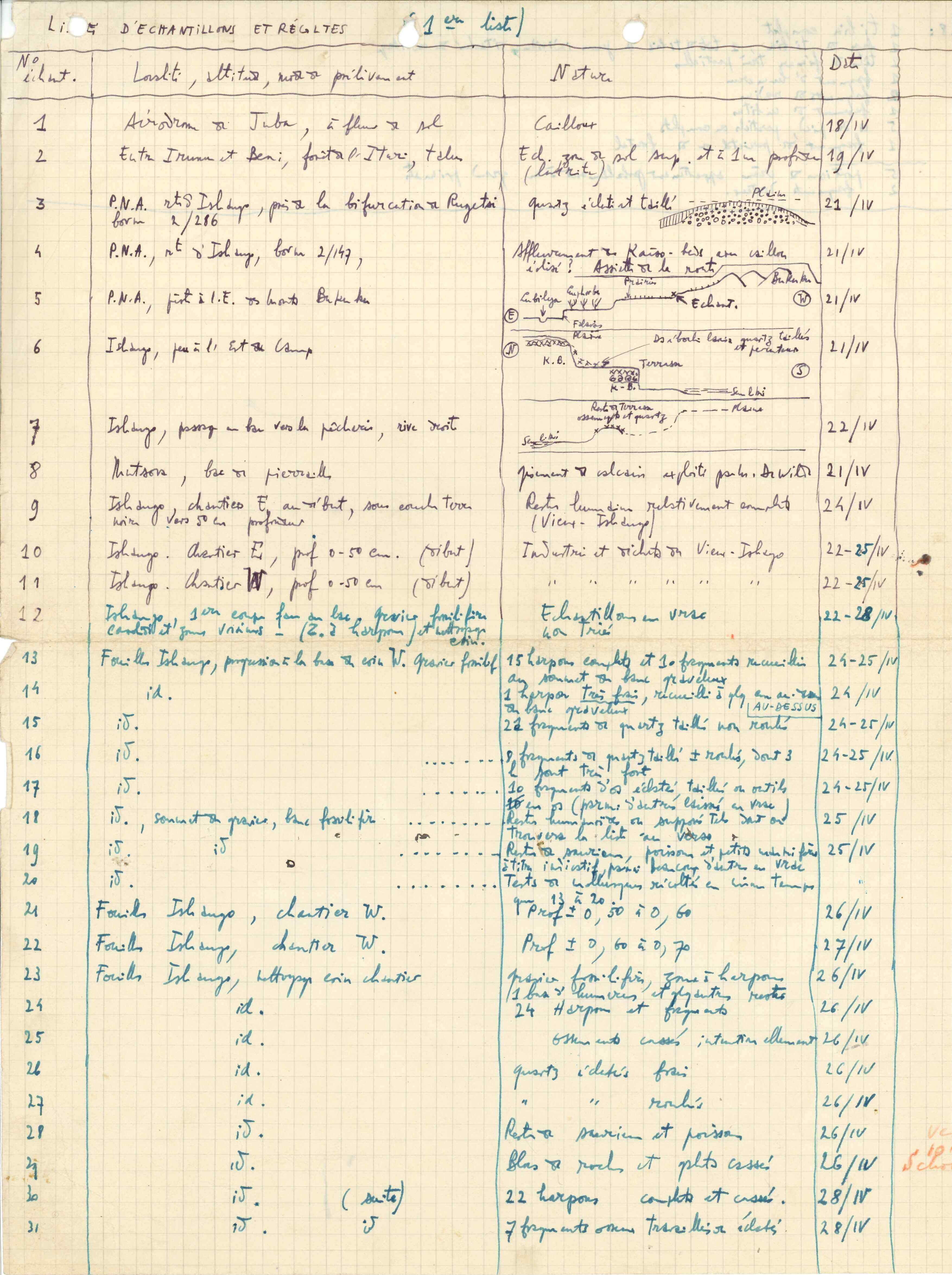
*

**Figure S5.** First page of the Heinzelin notebook. Verso.

"Liste d'échantillons et récoltes (1ère liste)

| *N° échant.* | *Localité, altitude, nbre de prélèvement* | *Nature* | *Date* |
| --- | --- | --- | --- |
| *(...)* | *(...)* | *(...)* | *(...)* |
| *9* | *Ishango, Chantier E au début, sous couche terre noire, vers 50 cm de profondeur* | *Restes humains relativement complets (Vieux-Ishango)* | *24/IV* |
| *10* | *Ishango. Chantier E, prof. 0-50cm (début)* | *Industrie et déchets du Vieux-Ishango* | *22-25/IV* |
| *11* | *Ishango. Chantier W, prof. 0-50cm (début)* | *Industrie et déchets du Vieux-Ishango* | *22-25/IV* |
| *12* | *Ishango, première coupe face au lac, gravier fossilifère consolidé et zones (v...?) - (Z. à harpons) et nettoyage coin* | *Echantillons en vrac*  *non triés* | *22-28/IV* |
| *13* | *Fouilles Ishango, progression à la base du coin W, gravier fossilif.* |  | *24-25/IV* |
| *(...)* | *(...)* | *(...)* | *(...)* |
| *18* | *Id., sommet du gravier, banc fossilifère* | *Restes humanoïdes ou supposés tels dont on trouvera la liste au verso* | *25/IV* |
| *(...)* |  | *(...)* | *(...)"* |

- First page of de Heinzelin notebook (Figure S5).

*List of the samples and pickings (Black ink) - (1st list) (Turquoise ink)*

| *N° sample* | *Locality, altitude, number of sampling* | *Nature* | *Date* |
| --- | --- | --- | --- |
| *(...)* | *(...)* | *(...)* | *(...)* |
| *9* | *Ishango, Beginning of E field, under the black layer of earth, around 50 cm depth* | *Human remains relatively complete (Old-Ishango)* | *24/IV* |
| *10* | *Ishango. E field, depth 0-50cm (beginning)* | *Industry and waster material from Old-Ishango* | *22-x/IV*  ("x" later corrected to 25 with Turquoise ink) |
| *11* | *Ishango. W field, depth 0-50cm (beginning)* | *Industry and waster material from Old-Ishango* | *22-x/IV*  ("x" later corrected to 25 with Turquoise ink) |
| *12* | (Start of use of the **Turquoise ink**) *Ishango, first section (...), fossiliferous gravel (...) - (Z. with harpoons) and cleaning corner* | *Unsorted samples* | *22-28/IV* |
| *13* | *Ishango excavations, progression at the base of W corner, fossiliferous gravels* |  | *24-25/IV* |
| *(...)* | *(...)* | *(...)* | *(...)* |
| *18* | *Id., summit of the gravel, fossiliferous level* | *Humanoid remains or supposed so, whose detailed list is given on back page* ([[1]](#footnote-2)) | *25/IV* |
| *(...)*([[2]](#footnote-3)) | *(...)* | *(...)* | *(...)* |

There is a change of ink in the written documents that occurs at the end of Monday 24th of April and this is observed coherently on both types of archives. Teeth are only mentioned in the letter to Twiesselmann and their discovery can be dated between the night of the 23^rd^ and the morning of the 25^th^ of April. The idea that the teeth mentioned could have been associated to a mandible fragment can be ruled out since there is no indication of a mandible in the list of sample N°18, while the base of the tibia is present. Therefore, there is strong evidence to suggest that the two isolated teeth discovered in the fossiliferous level at Ishango come from the human sample N°9, the only one without a clear description of its content. An alternative scenario is that the tooth was not recognized on site and identified later in the laboratory when the collection was in Belgium. However, it is unlikely for two reasons:

First, all human remains identified out in the field on the site were sent together to the Laboratory of Anthropology of the RBINS beginning of February 1951 (Figure S6).


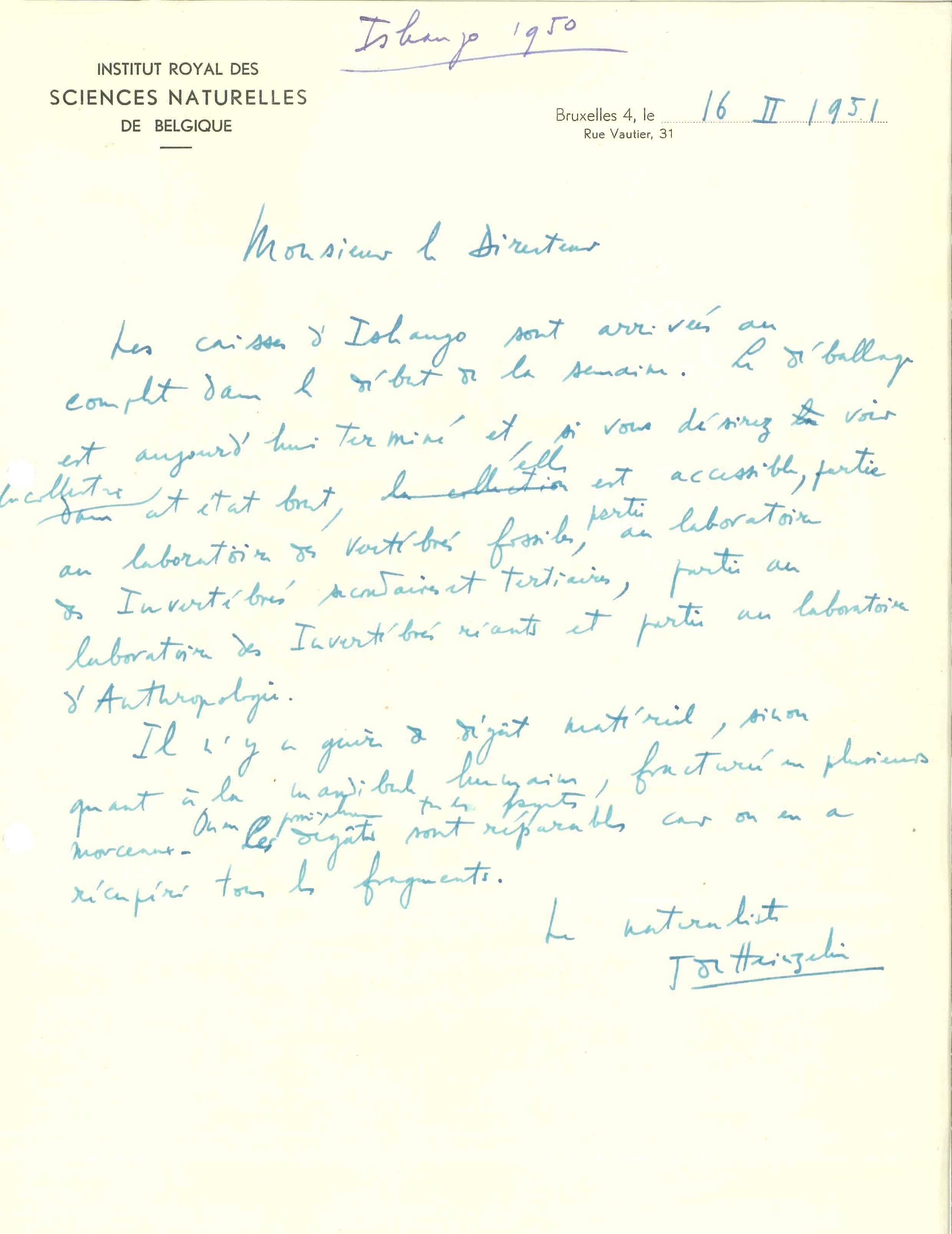


**Figure S6.** Letter from de Heinzelin to the director of the RBINS dated to the 16th of February 1951.

*"Ishango 1950. Institut royal des Sciences naturelles de Belgique. Bruxelles 4, le 16 II 1951 rue Vautier 31.*

*Monsieur le Directeur. Les caisses d'Ishango sont arrivées au complet dans le début de la semaine. Le déballage est aujourd'hui terminé et si vous désirez ~~la~~ voir la collection dans cet état brut, ~~la collection~~ elle est accessible, partie au laboratoire des vertébrés fossiles, partie au laboratoire des Invertébrés secondaires et tertiaires, partie au laboratoire des Invertébrés récents et partie au laboratoire d'Anthropologie. Il n'y a guère de dégât matériel, sinon quant à la mandibule humaine, facturée en plusieurs morceaux. On a possiblement tous les fragments. Ces dégâts sont réparables car on en a récupéré tous les fragments. Le naturaliste. J de Heinzelin."*

- Letter from de Heinzelin to the Director of the RBINS (Figure S6).

*Royal Belgian Institut of Natural sciences – Brussels 4, 16 II 1951 – Rue Vautier, 31*

*Mister the Director*

*The Ishango boxes arrived in full beginning of this week. The unpacking is now finished and if you would like to see the collection in its raw state, it is accessible, partly in the Laboratory of Vertebrate fossils, partly in the Laboratory of secondary and tertiary Invertebrates, partly in the Laboratory of recent Invertebrates and partly in the Laboratory of Anthropology.*

*There is no damage except for the human mandible, broken in several pieces. We possibly have all the fragments. The damages are reversible because we have collected all the fragments.*

*The naturalist,*

*J.de Heinzelin*

As mentioned in this letter (Figure S6), vertebrate and invertebrate fauna remains were separately stored in the different laboratories of the same institution right after their arrival in Belgium. Moreover, the inventory number on #Ish25 is identical to the other human remains, suggesting it was part of the same lot.

Second, the identification of the human remains made out in the field was extremely accurate and resulted in keeping small identifiable fragments, for instance perinatal remains. This lends support to the probability that isolated teeth would have been recognized right away.

This hypothesis in combination with the fact that most human remains were found during the first stages of the 1950-excavation in the western area, strongly argues for #Ish25 to have been found on April 24th, the second day of excavation, in the South-western part of the western corner (zone A in Figure S1). The first sketch of a stratigraphical section with the definition of the archaeological layers dates to May 9th 1950 (*cf.* Figure S7). The NFPr layer was at that time designated as GF (Fossiliferous Gravel), as written on the first pages of the notebook. The first map of the Ishango excavation dates from between the 5th to the 9^th^ of May 1950 and the western area is indicated as "*Progression of cleanup corner in the notebook until the 9/V*" (Figure S8). Since the area excavated the first days in the western corner only yielded the remains of the basal levels of the Ishango Gravels Formation (G.INF and the basal part of NFPr) and that the stratigraphy was not yet clearly established the 24th of April, it can be argue that #Ish25 was found either in G.INF or in the NFPr.

**
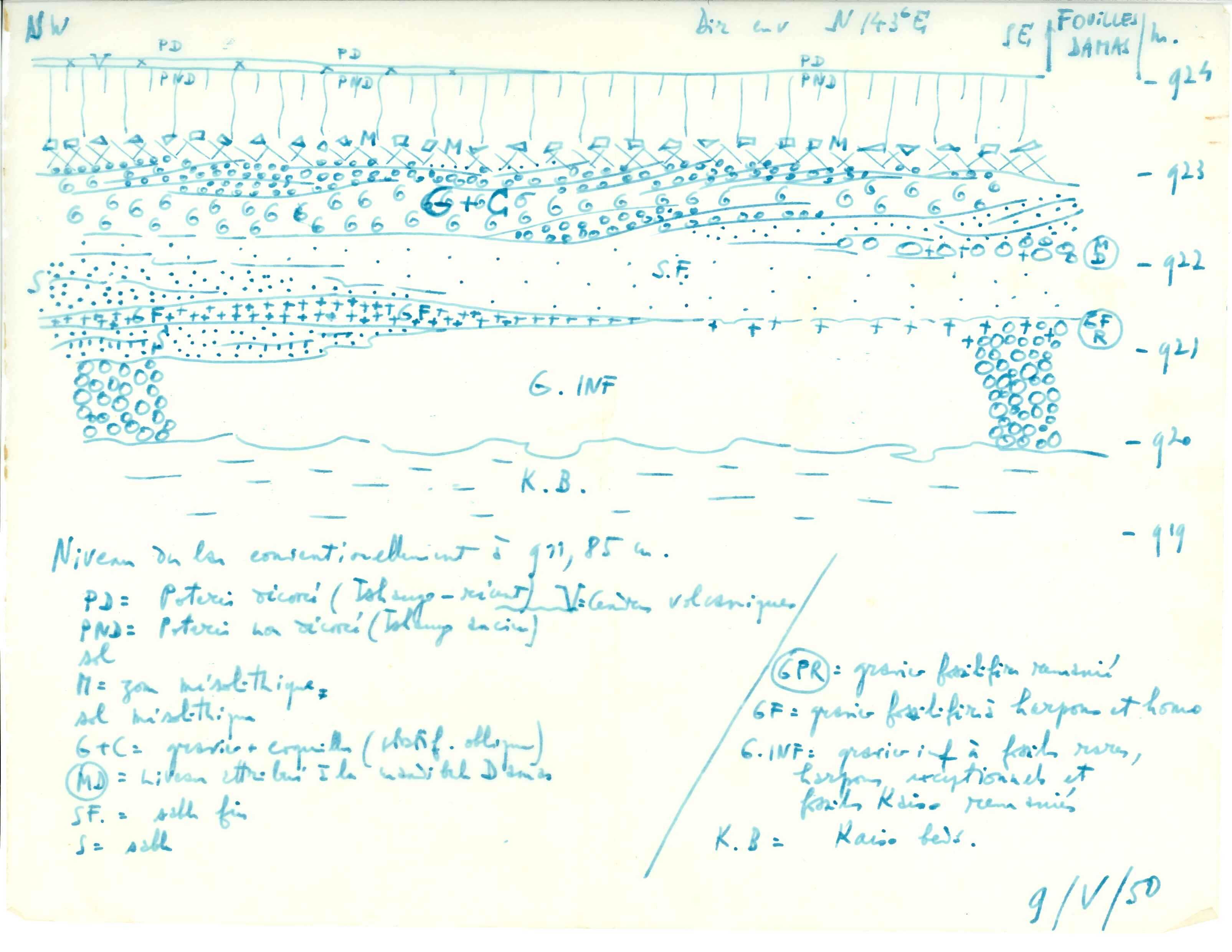
**

**Figure S7.** First sketch of a stratigraphical section with the definition of the archaeological layers.

It is dated to the 9th May 1950. *"(...) GF = gravier fossilifère à harpons et homo. G.INF = gravier inf à fossiles rares, harpons exceptionnels et fossiles Kaiso remaniés. K.B = Kaiso beds. 9/V/50"*

*
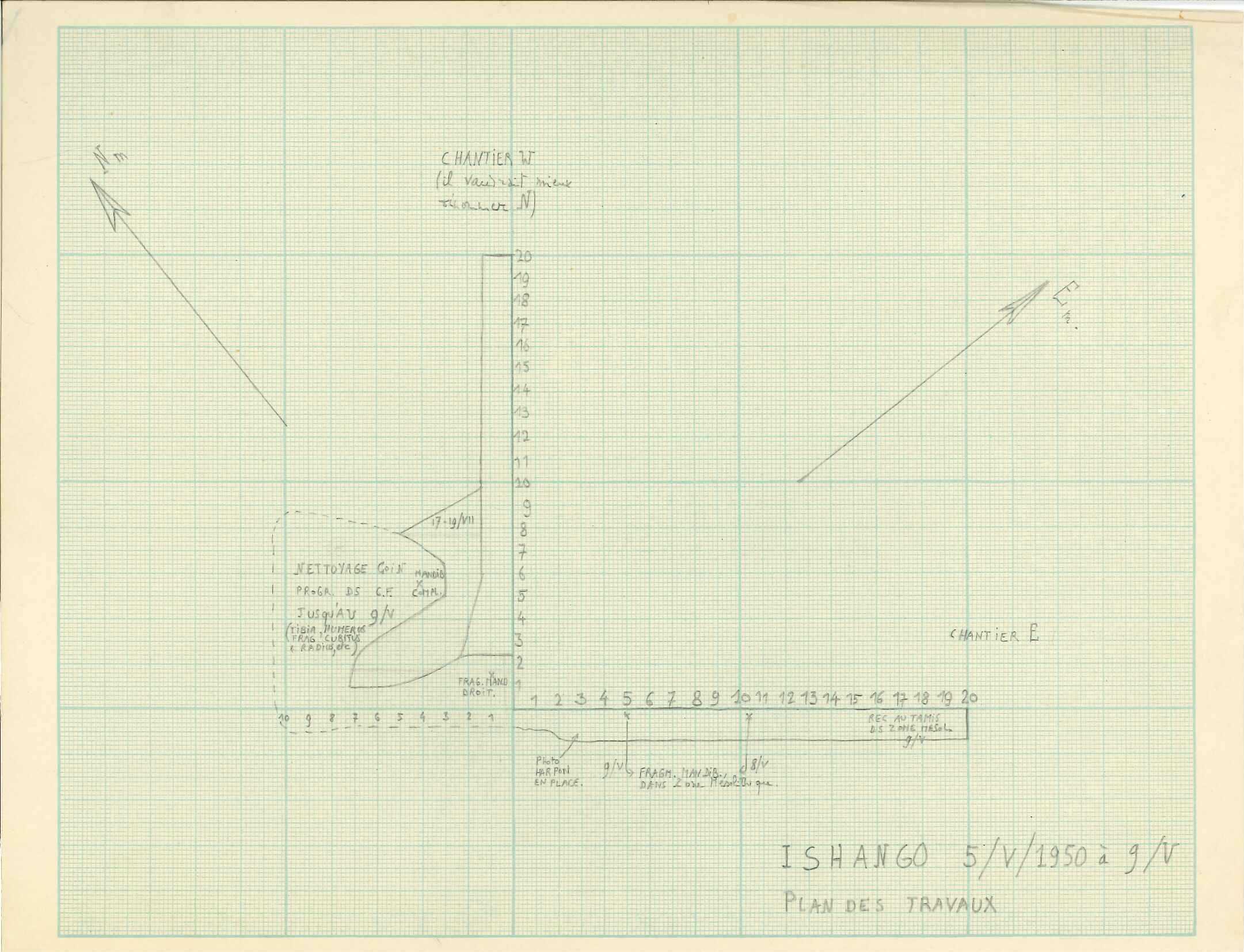
*

**Figure S8.** First map of the Ishango excavation dated from the 5th to 9th of May 1950.

*"CHANTIER W (il vaudrait mieux dénommer N) - 17-19/VII - NETTOYAGE COIN - mandib x compl. - Progr. ds G.F. jusq'au 9/V (tibia, humerus frag cubitus radius, etc) (...) ISHANGO 5/V/1950 à 9/V - Plan des travaux"*

**Text S3. Raman spectroscopic analysis**

As a calcified tissue, dentine is composed of approximately 70% of inorganic mineral (crystalline hydroxylapatite, Ca_10_(PO_4_)_6_(OH)_2_) and 30% of organic matter (collagen, CH). As soon as diagenetic processes take place, the interaction with the environment (*i.e.* sedimentary matrix) leads to biodegradation of the organic matter contained in the teeth and to the integration of new inorganic compounds like sulphates or carbonates of calcium and magnesium according to the environmental deposit chemistry [38]. Because its structure is more porous, dentine is more prone to be altered by diagenesis than enamel [39]. Person *et al.* [40-41] have shown that the composition and structural changes of fossil bone carbonate hydroxylapatite occurs early in the diagenetic process and depends on the burial environment. They observed a correlation between the increase in crystallinity and the decrease of CO_3_ amount, but not with the preservation of the organic matter. Thus, diagenetic changes in the dentine are independent of the age of the sample, they only relate to the taphonomic conditions [40-42].

We selected three well-preserved teeth from the NFPr layer (#Ish16, #Ish22 & #Ish24) that best represented the observable range of taphonomical alteration in the LSA sample (Figure S9). Moreover, #Ish24 is the only other isolated maxillary tooth from the site, supposedly discovered the same day as #Ish25. Then, we applied the same procedure as for #Ish25 to test, based on the Raman response, whether all teeth experienced the same taphonomic history. Several spectra (~ 10) were collected on the dentine surface of each tooth and the average results are given in the Figure 3. The Raman bands in the range of 1200-3000 cm^-1^ are mainly related to the organic matter (protein component, CH) [43]. No peaks are observable in this section of the spectra.


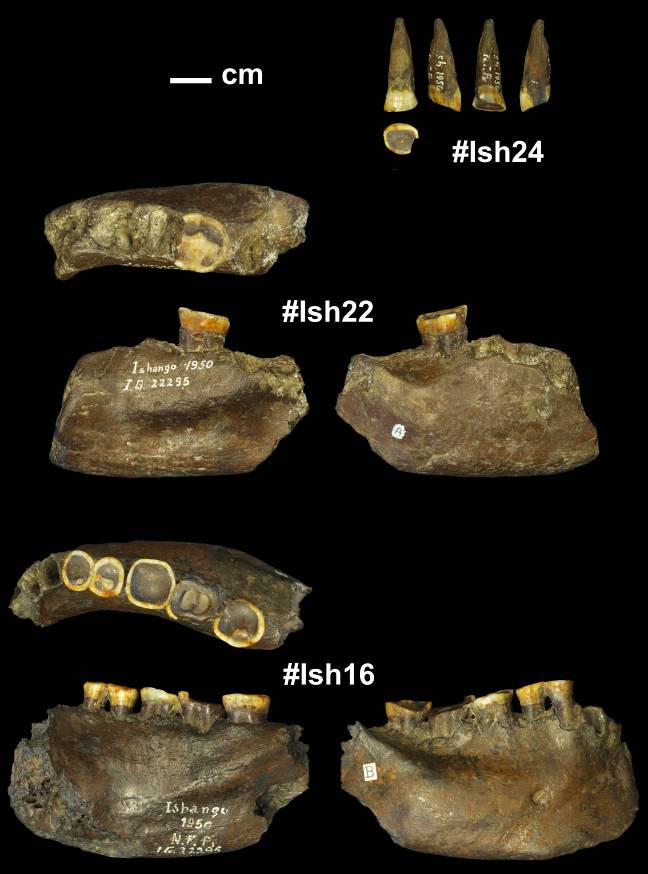
Although the Lusso Formation has yielded numerous fossils in the Semliki valley, at Ishango these layers that lie beneath the G.INF level have not been excavated. The reworked Lusso fossils found in the TT Formation at Ishango were shells, mammal or fish bones. Therefore, no teeth unambiguously associated to the Lusso beds were available for comparable analysis.

**Figure S9.** Photographs of the teeth from the LSA human assemblage (NFPr level) that were used as comparative samples for the Raman spectroscopic analyses.

From left to right, top to bottom: #Ish24= left central upper incisor, buccal, lateral, lingual, medial and occlusal views; #Ish22=fragment of a right mandibular corpus with the second lower molar included, occlusal, lingual and buccal views; #Ish16= right hemi-mandible with the two lower premolars and three lower molars included, occlusal, lingual and buccal views. In the latter case, the analysis was performed on the right first lower molar.

**Text S4. Morphometric comparison**

Text S4.1 Crown dimensions

The exceptional mesio-distal and vestibulo-lingual dimensions of #Ish25 M^1^ crown were first noticed by Twiesselmann in 1958 in his monograph ("*Les dimensions considérables de la dent d'Ishango rejoignent celles des fossiles de Swartkrans; son gabarit humanoïde la rapproche, autant que j'en puisse juger, de la dent de I'Australopithèque de Taungs; trouvée isolément, elle aurait pu conduire à supposer une parenté rapprochée avec les australopithèques*"; [23], p.89), then by Orban *et al.* [44]. Table S1 gives the crown dimensions of Ishango M^1^ as well as the mean and range of variation of the comparative groups. Figure S10 represents the scatterplot of the mesiodistal and buccolingual M^1^ diameters of #Ish25 and the comparative sample. For better graphic readability, and because overlap in crown dimensions has been stated by various authors [45-47], we grouped together all the gracile and robust Australopiths M^1^ dimensions.

Measurements are compiled from published data [48-122] and from personal measurements on original fossils from B.M. (Monsempron, Petit-Puymoyen) and I.C. on original fossils (Taforalt, Jebel Sahaba, Lothagam, Wadi Halfa & NEO).

Adjusted Z-Scores [123] were computed to compare the dimensions of the #Ish25 to the mean and standard deviations of the comparative groups.

**Table S1:** Crown dimensions (mm) of #Ish25 and the comparative group means and standard deviations

|  | #Ish25 | AUSGR* | EH | HERG | HROD | HHEID | ATER | NEAN | MPHS | UPHS | LUPA | NEO | RMH |
| --- | --- | --- | --- | --- | --- | --- | --- | --- | --- | --- | --- | --- | --- |
| MD^#^ |  |  |  |  |  |  |  |  |  |  |  |  |  |
| mean | 13.10 | 13.12 | 13.16 | 12.45 | 11.44 | 11.65 | 11.60 | 11.17 | 11.37 | 10.86 | 11.08 | 10.82 | 9.64 |
| stdev |  | 1.10 | 0.80 | 0.44 | 0.82 | 0.54 |  | 0.85 | 0.55 | 0.73 | 0.61 | 0.61 | 0.97 |
| n |  | 83 | 23 | 4 | 7 | 11 | 2 | 50 | 31 | 93 | 85 | 123 | 538 |
| Adjusted Z-Scores | | -0.008 | -0.038 |  | 0.772 | **1.158** |  | **1.110** | **1.514^¤^** | **1.523^¤^** | **1.659^¤^** | **1.871^¤^** | **1.806^¤^** |
| BL^#^ |  |  |  |  |  |  |  |  |  |  |  |  |  |
| mean | 14.20 | 14.31 | 13.14 | 12.87 | 12.26 | 12.05 | 13.25 | 12.09 | 12.06 | 12.24 | 12.26 | 11.86 | 10.95 |
| stdev |  | 1.23 | 0.72 |  | 0.93 | 0.73 |  | 0.71 | 0.65 | 0.75 | 0.57 | 0.62 | 0.68 |
| n |  | 80 | 22 | 3 | 8 | 11 | 2 | 52 | 30 | 94 | 85 | 128 | 535 |
| Adjusted Z-Scores | | -0.045 | 0.693 |  | 0.647 | **1.268** |  | **1.455^¤^** | **1.570^¤^** | **1.309** | **1.699^¤^** | **1.879^¤^** | **2.412^¤^** |
| BL/MD |  |  |  |  |  |  |  |  |  |  |  |  |  |
| mean | 108.4 | 109.00 | 99.79 | 103.40 | 110.10 | 103.40 | 121.00 | 108.60 | 106.40 | 112.90 | 110.80 | 110.00 | 114.40 |
| stdev |  | 5.79 | 4.28 |  | 7.13 | 4.39 |  | 7.88 | 8.13 | 5.79 | 5.72 | 5.33 | 10.08 |
| n |  | 79 | 22 | 3 | 7 | 11 | 2 | 49 | 30 | 93 | 85 | 123 | 531 |
| Adjusted Z-Scores | | -0.051 | 0.946 |  | -0.099 | 0.489 |  | -0.015 | 0.118 | -0.394 | -0.207 | -0.152 | -0.304 |

*AUSGR: Gracile and Robust Australopiths from Afar, Bouri, Chesowanja, Drimolen, Kanapoi, Kokiselei, Koobi Fora, Kromdraai, Lomekwi, Makapansgat, Olduvai Gorge, Omo Valley, Sterfontein, Swartkrans, Taung; EH: Early *Homo* (*Homo habilis, Homo rudolfensis, Homo georgicus)* from Afar, Cornelia-Uitzoek, Dmanisi, Drimolen, Koobi Fora, Olduvai Gorge, Omo Valley, Swartkrans; HERG: *Homo ergaster* (KNM-WT 15000, KNM-ER807, KNM-ER3733; KGA11-350); HROD: *Homo rhodesiensis* (Middle Pleistocene *Homo* from Africa including Eyasi1, Kabwe (Broken Hill), Laetoli H18, Rabat 1 & 2, Salé, Ternifine (Tighenif)); HHEID: *Homo heidelbergensis* (Middle Pleistocene *Homo* from Europe including Arago, Bilzingsleben, Petralona, Sima de los Huesos, Steinheim); ATER: Specimens associated to Aterian levels in North Africa (Dar-es-Soltan II (H4) & El Haroura); NEAN: Specimens attributed to Neandertal lineage from Eurasia (Amud, Arcy, Baume Néron, Châteauneuf, Combe Grenal, Cotencher, Hortus, Krapina, Kulna, La Chaise, La Côte Sainte-Brelade, La Ferrassie, La Quina, Le Moustier, Monsempron, Petit-Puymoyen, Pontnewydd, Saccopastore, Shanidar, Subalyuk, Teshik-Tash, Visogliano); MPHS: Middle Pleistocene/Middle Palaeolithic *Homo sapiens* from Africa and Southwest Asia (Herto, Qafzeh, Skhul); UPHS: Upper Pleistocene/Upper Palaeolithic *Homo sapiens* from Europe and Africa (Abri Pataud, Arene Candide, Cap-Blanc, Dolni vestonice, Fontéchevade, Gazel, La Crouzade, Lagar Velho, La Madeleine, Les Hoteaux, Mladec, Montgaudier, Muierii, Nazlet Khater, Paglicci, Pavlov, Pestera cu Oase, Pont d'Ambon, Predmosti, Roc de Cave, Romiton, Saint-Germain, San Teodoro, vado All'Arancio); LUPA: Late Upper Pleistocene-Early Holocene populations from Africa (Jebel Sahaba, Lothagam, Taforalt, Wadi Halfa); NEO: Neolithic populations from Europe and Africa; RMH: Recent modern humans. **^#^**Measurements from Martin [124]; MD = Mesio-distal diameter of the crown (M.81); BL = Bucco-lingual diameter of the crown (M.81(1)). m: mean; s: standard deviation; n: number. Adjusted Z-scores (0: mean; +1/-1: upper and lower limits of the comparative group at 95%). The values in bold represent a probability less than 0.05 for #Ish25 to belong to the comparative sample. **^¤^**p < 0.01.


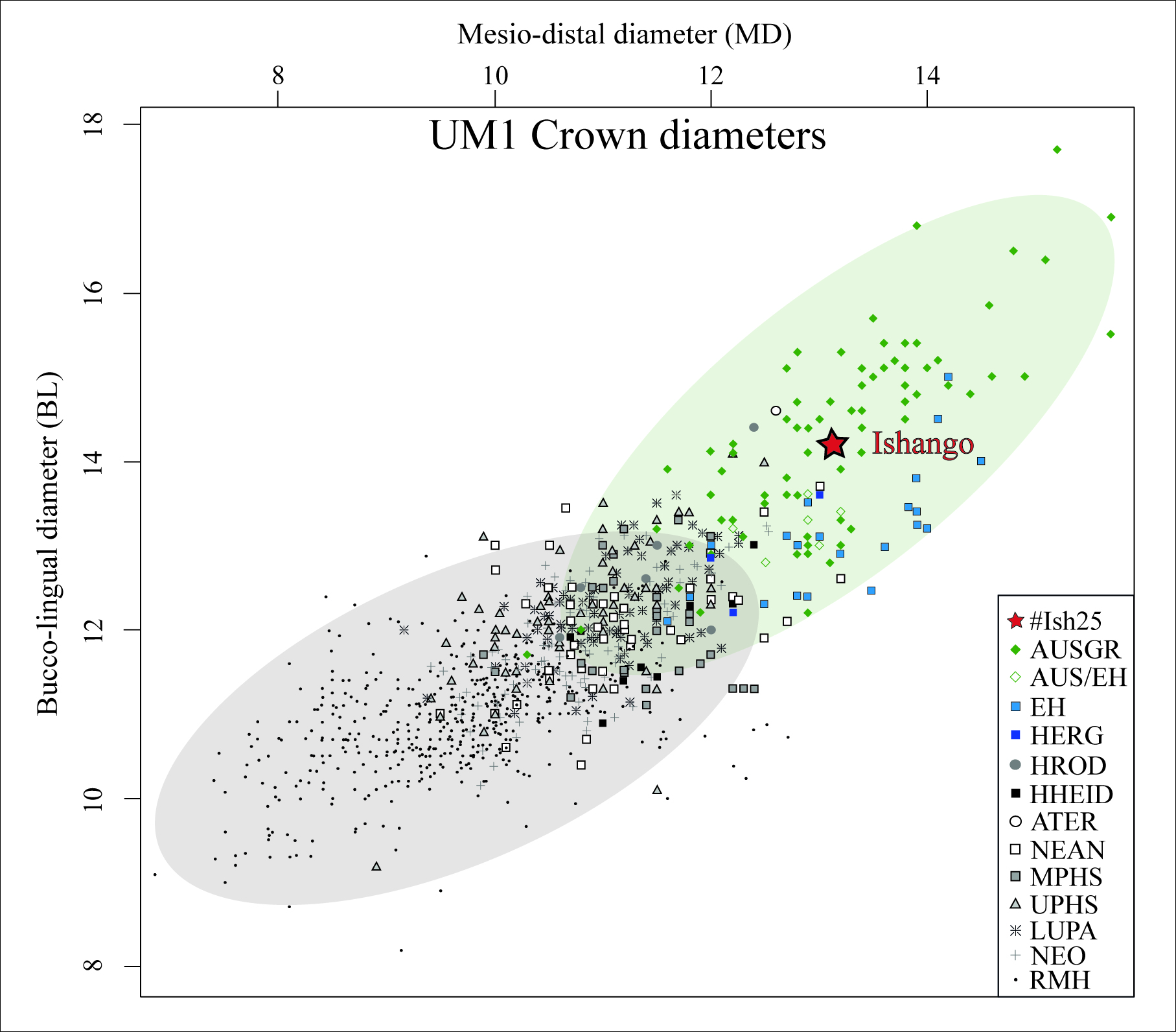


**Figure S10.** Bivariate plot of the crown diameters of #Ish25 and the comparative sample (*cf.* Table S1).

Text S4.2 Cusp area analysis

The cusp area proportions of #Ish25 were assessed following the method described by Quam *et al.* [125]. However, given the complexity of the mesial marginal ridge with its two large mesial accessory cuspules, the cusp area analysis of #Ish25 is somewhat ambiguous. Following the standard protocol, the division was made to include one in each the protocone and the paracone, with the largest cuspule attributed to the protocone.

Values of #Ish25 M^1^ and comparative sample means are displayed in the Table S2. The crown base area of #Ish25 is large (158.2 mm^2^), falling between the mean of gracile (150.6 mm^2^) and robust (161.5 mm^2^) australopithecines, and at the high end of the range for early *Homo* ($\bar{X}$= 135.5 mm^2^, range 161.5-110.4 mm^2^). The cusp areas of the Ishango molar have the following relationships: protocone > paracone > metacone > hypocone. In nearly all *Australopithecus* and *Paranthropus* the paracone was between 85% and 95% the size of the metacone. Most of the early *Homo* also exhibited this presumed primitive (metacone > paracone) pattern. In contrast, in nearly all later *Homo* fossils the paracone was found to be larger than the metacone (5%-27% larger). This derived pattern was observed in 83.3% of the *Homo erectus* sample, 100% of the Neandertal sample and 85.7% of the Middle Pleistocene *H. sapiens* sample (note the remaining 14.3% of the Middle Pleistocene *H. sapiens* sample had equally sized paracone and metacone, not the primitive condition). #Ish25 M^1^ possesses the derived pattern with a paracone nearly 5.5% larger than the metacone. This places it to the right of the diagonal line representing equal sized paracone and metacone (Figure S11), in the later *Homo* conformation. The derived condition was also observed in the robust australopithecine sample (3%; one individual) and in the early *Homo* group (11.1%; OH39 and KNM-ER 1813) while one individual from the gracile australopithecine sample had cusps of equal size within 1% of each other) (see [125]).

A principal components analysis of relative cusp areas shows a great deal of variation both within and between hominin species (Figure S12). PC1 (43.3% of the variation) primarily separates individuals with the primitive (negative scores) versus derived (positive scores) metacone/paracone relationship. PC2 is more difficult to interpret, but separates, at least to some extent, those individuals with a relatively large hypocone from those with a relatively small hypocone. #Ish25 has positive scores for PC1 and PC2. Other individuals with positive scores for PC1 and PC2 include primarily Middle Pleistocene and Upper Paleolithic *H. sapiens*, which possess a smaller metacone relative to paracone and a smaller hypocone relative to metacone.

**Table S2:** Comparison of crown and cusp areas between Ishango (#Ish25) and comparative fossil groups (mm^2^).

| GROUPS* | TCA | PROTO | PARA | META | HYPO | PROTO% | PARA% | META% | HYP% | PAR/MET |
| --- | --- | --- | --- | --- | --- | --- | --- | --- | --- | --- |
| #Ish25 | 158.2 | 49.3 | 38.7 | 36.63 | 33.6 | 31.1 | 24.4 | 23.2 | 21.3 | 108.3 |
|  |  |  |  |  |  |  |  |  |  |  |
| SAFGRA (n=7) | 150.6 | 45.9 | 31.1 | 36.6 | 36.9 | 30.5 | 20.7 | 24.45 | 24.3 | 85.25 |
| EAFGRA (n=3) | 150.9 | 49.6 | 34.05 | 36.0 | 31.3 | 32.8 | 22.6 | 23.8 | 20.7 | 95.3 |
| ***PooledGRA*** | ***150.6*** | ***46.7*** | ***31.7*** | ***36.2*** | ***35.7*** | ***31.0*** | ***21.2*** | ***24.3*** | ***23.5*** | ***87.5*** |
|  |  |  |  |  |  |  |  |  |  |  |
| SAFROB (n=17) | 156.7 | 45.1 | 34.7 | 39.9 | 37.1 | 28.8 | 22.1 | 25.4 | 23.6 | 87.4 |
| EAFROB (n=3) | 188.65 | 56.7 | 41.1 | 46.4 | 44.1 | 30.1 | 21.8 | 24.6 | 23.3 | 88.65 |
| ***PooledROB*** | ***161.5*** | ***46.8*** | ***35.6*** | ***40.9*** | ***38.1*** | ***29.0*** | ***22.1*** | ***25.3*** | ***23.6*** | ***87.6*** |
|  |  |  |  |  |  |  |  |  |  |  |
| SAFEH (n=4) | 144.7 | 43.1 | 34.4 | 35.4 | 31.8 | 29.7 | 23.8 | 24.5 | 22.1 | 97.1 |
| EAHEH (n=14) | 132.9 | 38.6 | 30.2 | 33.3 | 30.9 | 29.1 | 22.8 | 25.4 | 23.1 | 91.5 |
| ***PooledEH*** | ***135.5*** | ***39.6*** | ***31.1*** | ***33.7*** | ***31.1*** | ***29.2*** | ***23.0*** | ***24.9*** | ***22.8*** | ***92.8*** |
|  |  |  |  |  |  |  |  |  |  |  |
| HANT (n=4) | 125.4 | 37.2 | 31.0 | 26.45 | 30.75 | 29.7 | 24.75 | 21.1 | 24.5 | 119.4 |
| HER S.L. (n=6) | 112.8 | 33.7 | 28.45 | 26.4 | 24.3 | 29.9 | 25.3 | 23.4 | 21.5 | 108.9 |
| HHEID (n=5) | 115.8 | 34.9 | 28.9 | 23.9 | 28.1 | 30.9 | 25.1 | 20.0 | 23.9 | 126.6 |
| NEAN (n=21) | 112.3 | 33.7 | 28.8 | 22.9 | 26.8 | 29.9 | 25.8 | 20.6 | 23.7 | 126.7 |
| MPHS (n=7) | 111.3 | 34.7 | 27.5 | 23.7 | 25.8 | 31.3 | 24.8 | 21.3 | 22.8 | 117.6 |
| UPHS (n=18) | 103.3 | 32.7 | 25.8 | 23.3 | 21.5 | 32.2 | 25.75 | 22.7 | 20.8 | 115.2 |
|  |  |  |  |  |  |  |  |  |  |  |
| **Adjusted Z-Scores** |  |  |  |  |  |  |  |  |  |  |
| Ish - GRA | 0.2 | 0.4 | **1.2** | 0.1 | -0.1 | 0.0 | 0.9 | -0.2 | -0.3 | 0.9 |
| Ish - ROB | -0.1 | 0.2 | 0.3 | -0.4 | -0.3 | 0.5 | 0.6 | -0.6 | -0.5 | 0.9 |
| Ish - EH | 0.7 | 0.8 | 0.9 | 0.3 | 0.2 | 0.4 | 0.3 | -0.4 | -0.3 | 0.6 |
| Ish - LH | **1.4** | **1.4** | **1.4** | **1.5** | 0.7 | 0.0 | -0.2 | 0.3 | -0.2 | -0.3 |

*SAFGRA: South African Gracile Australopiths, EAFGRA: East African Gracile Australopiths, SAFROB: South African Robust Australopiths, EASROB: East African Robust Australopiths, SAFEH: South African early *Homo*, EASEH: East African early *Homo*, HANT: *Homo* *antecessor*, HER S.L.: *Homo erectus* sensu lato (*H. ergaster*, n=1; Asian *Homo erectus*, n=5), HHEID: *Homo heidelbergensis*; NEAN: Neandertals, MPHS: Middle Pleistocene *H. sapiens*, UPHS: Upper Paleolithic *H. sapiens*; LH: Late *Homo.* Details of the sample composition can be found in Quam *et al.* [125]. Four *H. antecessor* individuals (ATD6-10, ATD6-69,ATD6-103 and ATD6-11), one *H. heidelbergensis* specimen (Rabat), and three Upper Paleolithic *H. sapiens* specimens (Cisterna, Sunghir 2 and Sunghir 3) were added for this study. TCA: Total cusp area; PROTO: protocone; PARA: paracone, META: metacone; HYPO: hypocone. n: number. Adjusted Z-scores (*cf.* Table S1).


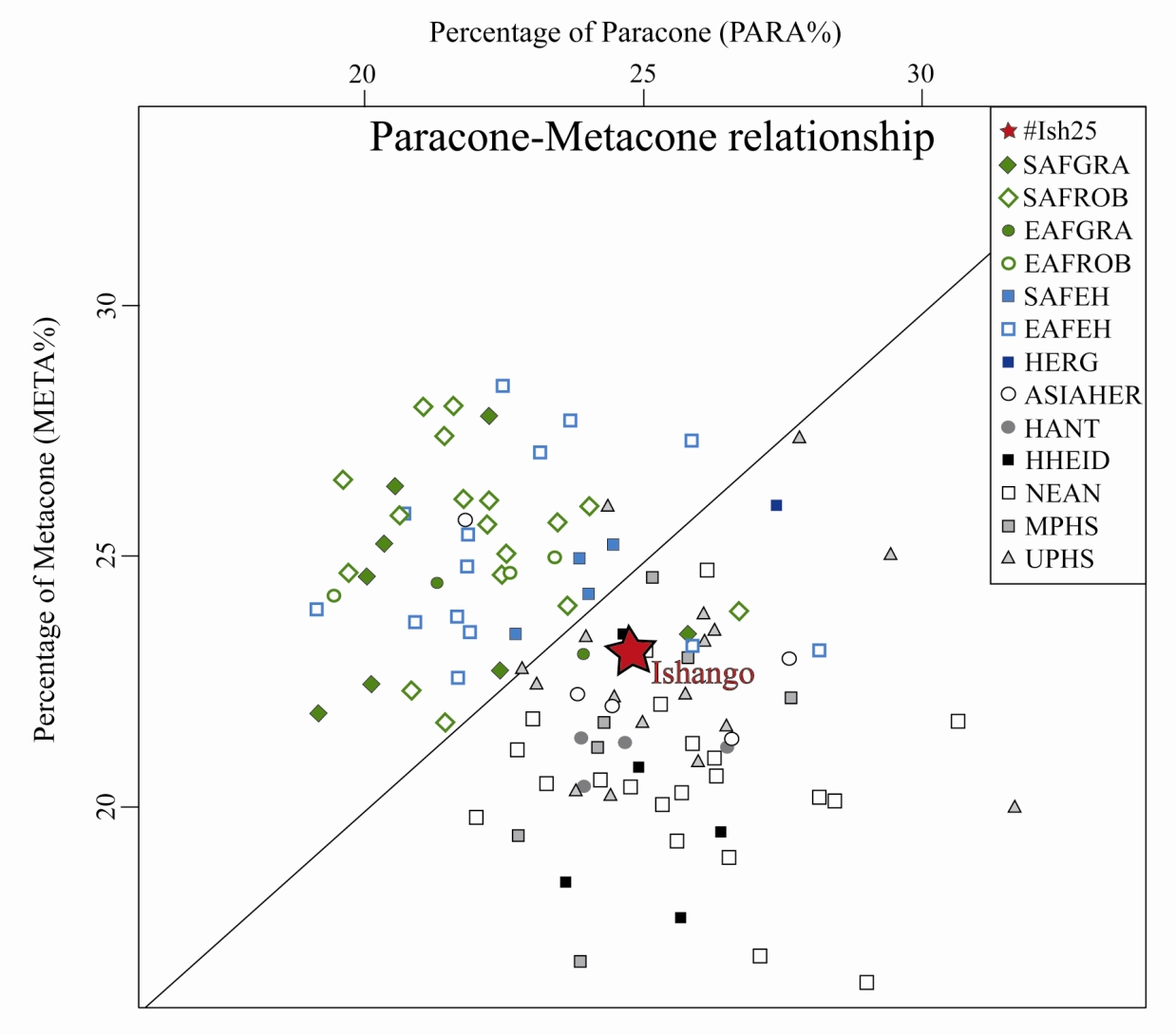


**Figure S11.** Bivariate plot of the relative paracone area in relation to the relative metacone area. Comparative samples as in Table S2.


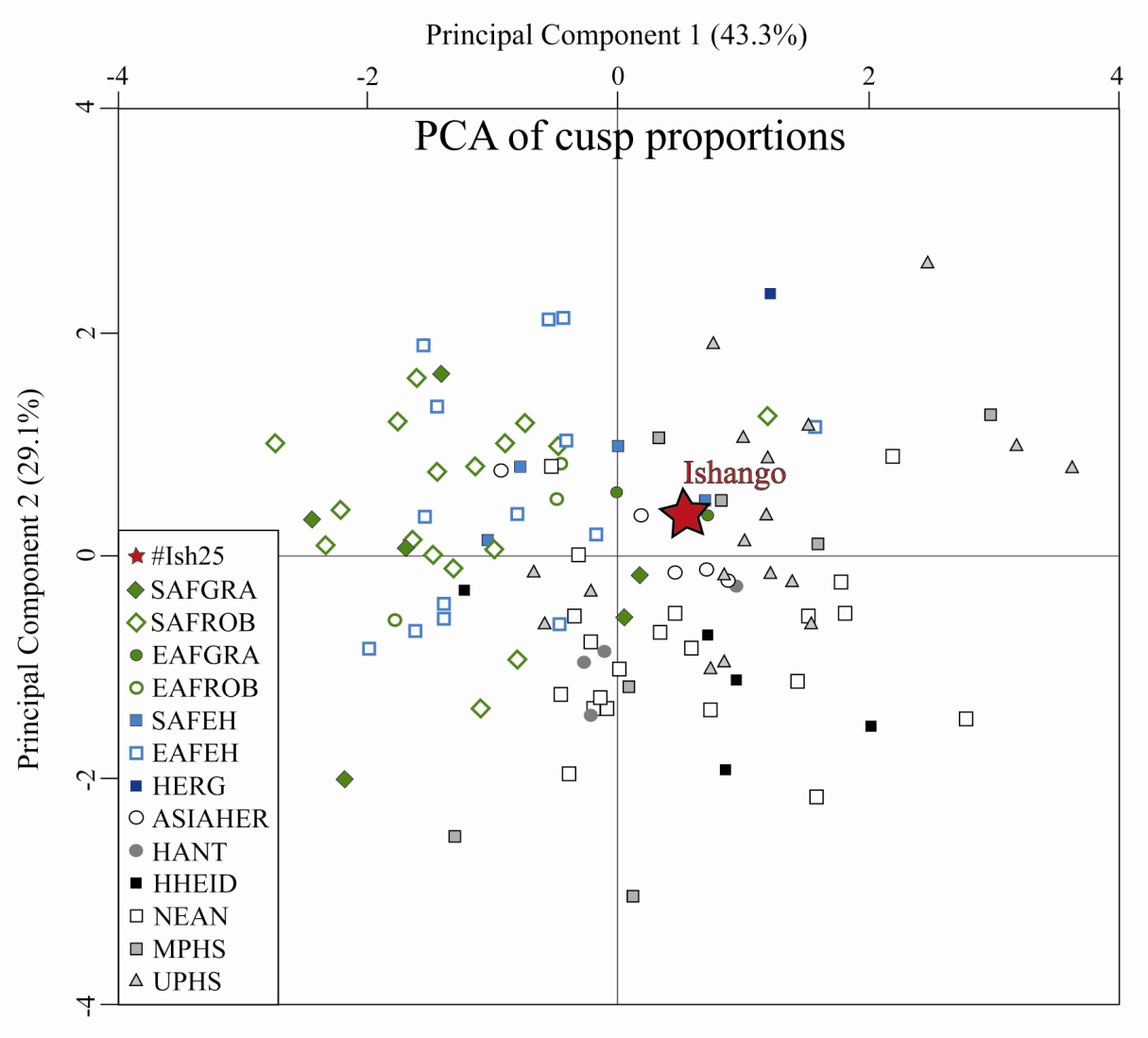


**Figure S12.** Scatter plot of the first and second principal components of the PCA on relative cusp areas. Comparative samples as in Table S2.

Text S4.3 Two- and three-dimensional dental tissue proportions

Micro-computed tomography allows the non-destructive two- and three-dimensional analyses of dental tissue proportions of fossil teeth that cannot be physically sectioned (*e.g.* [126-128]). The enamel and dentine crown proportions of #Ish25 are given in Table S3 and Table S4 with the mean and range of variation of the comparative samples. The values of the closest first upper molar to #Ish25 in the Figure S14 (*Australopithecus africanus*, Sts 57) is displayed for information in the Table S4.

In Figure S13, three variables stand outside the upper limit of the recent modern human variation (A, the total area of the tooth crown section; B, the area of dentine under the enamel cap; E, the perimeter length of the enamel-dentine junction). #Ish25 clusters with early hominins from East and southern Africa regarding the raw values (A to E). The relative enamel thickness (RET2D) of #Ish25 attests to thicker enamel than the modern human average, as it has been characterized for *Australopithecus* and *Paranthropus* [129-131], but lies within the modern human range of variation range, like Sts 57 (dashed green line). The specimens of Middle to Upper Pleistocene *Homo* are closer to the modern human proportions as underlined by Smith *et al.* [132].

The Figure S14 illustrates the significant negative correlation between the relative enamel thickness and the coronal dentine and pulp volume [133], showing that reduced dental size implies thicker enamel by millimeter units in the modern human sample. The position of #Ish25 is characterized by a proportionally high enamel thickness compared to modern humans. In this respect, it lies close to two early hominins M^1^s from Sterkfontein (Sts 57) and Swartkrans (SK 832).

**Table S3:** Two-dimensional dental tissue proportions of M^1^ #Ish25 and the first upper molar comparative samples.

|  | A*  (mm^2^) | C  (mm^2^) | B  (mm^2^) | E  (mm) | BCD  (mm) | AET2D  (mm) | RET2D |
| --- | --- | --- | --- | --- | --- | --- | --- |
| #Ish25 | 84.89 | 32.34 | 52.55 | 22.27 | 13.8 | 1.45 | 20.03 |
| SAFGRA |  |  |  |  |  |  |  |
| Sts 57 | 80.54 | 33.93 | 46.61 | 22.05 | - | 1.54 | 22.54 |
| RMH |  |  |  |  |  |  |  |
| m | 64.32 | 23.23 | 41.09 | 18.52 | 11.19 | 1.18 | 17.65 |
| s | 8.69 | 5.30 | 5.03 | 1.06 | 0.52 | 0.26 | 3.99 |
| n | 18 | 18 | 18 | 15 | 31 | 16 | 18 |
| Adjusted Z-Scores | |  |  |  |  |  |  |
| Ish-RMH | **1.092** | 0.793 | **1.051** | **1.596** | **2.423^¤^** | 0.477 | 0.275 |

*Measurement definitions following Martin [134], A: total area of the tooth crown section; C : area of the enamel cap; B : area of the dentine under the enamel cap enclosed by the enamel-dentine junction and the bi-cervical diameter; E : perimeter length of the enamel-dentine junction between the buccal and lingual cervical margins; BCD : bi-cervical diameter; AET2D: average enamel thickness; RET2D: scale-free relative enamel thickness. SAFGRA: *Australopithecus africanus* (Sterkfontein, [131]); RMH: Recent modern humans (pooled data, [134-136]). m: mean; s: standard deviation; n: number. Adjusted Z-scores values in bold represent a probability inferior to 0.05 for #Ish25 to belong to the comparative sample. **^¤^**p < 0.01.

**Table S4:** Three-dimensional dental tissue proportion of M^1^ #Ish25 and the pooled upper molar comparative samples.

|  | CVOL*  (mm^3^) | EVOL  (mm^3^) | DPVOL  (mm^3^) | EDJsa  (mm^2^) | AET3D  (mm) | RET3D |
| --- | --- | --- | --- | --- | --- | --- |
| #Ish25 | 869.03 | 414.22 | 454.81 | 271.04 | 1.53 | 19.87 |
| SAFGRA |  |  |  |  |  |  |
| Sts 57 | 873.37 | 382.29 | 491.08 | 240.58 | 1.59 | 20.14 |
| NEAN |  |  |  |  |  |  |
| m | 570.08 | 232.14 | 337.94 | 220.73 | 1.06 | 15.27 |
| s | 69.84 | 22.20 | 54.80 | 23.37 | 0.09 | 1.76 |
| n | 10 | 10 | 10 | 10 | 10 | 10 |
| RMH |  |  |  |  |  |  |
| m | 427.78 | 211.77 | 216.00 | 158.08 | 1.41 | 24.31 |
| s | 89.16 | 36.67 | 63.48 | 47.90 | 0.29 | 6.69 |
| n | 24 | 24 | 24 | 24 | 24 | 24 |
| Adjusted Z-Scores | |  |  |  |  |  |
| Ish-NEAN | **1.804^¤^** | **3.457^¤^** | 0.899 | 0.907 | **2.201^¤^** | **1.102** |
| Ish-RMH | **2.344^¤^** | **2.615^¤^** | **1.782^¤^** | **1.117** | 0.196 | -0.314 |

*Measurement definitions following Kono [135] & Olejniczak [137]. CVOL: volume of the crown; EVOL: volume of the enamel cap; DPVOL: volume of the crown dentine & pulp; EDJsa: surface area of the enamel-dentine junction; AET3D: the average enamel thickness; RET3D: the scale-free relative enamel thickness.

SAFGRA: *Australopithecus africanus* (Sterkfontein); NEAN: Specimens attributed to Neandertal lineage from Eurasia (Engis, Le Moustier, Scladina, El Sidron); RMH: Recent modern humans. m: mean; s: standard deviation; n: number. Adjusted Z-scores values in bold represent a probability inferior to 0.05 for #Ish25 to belong to the comparative sample. **^¤^** p < 0.01.

**
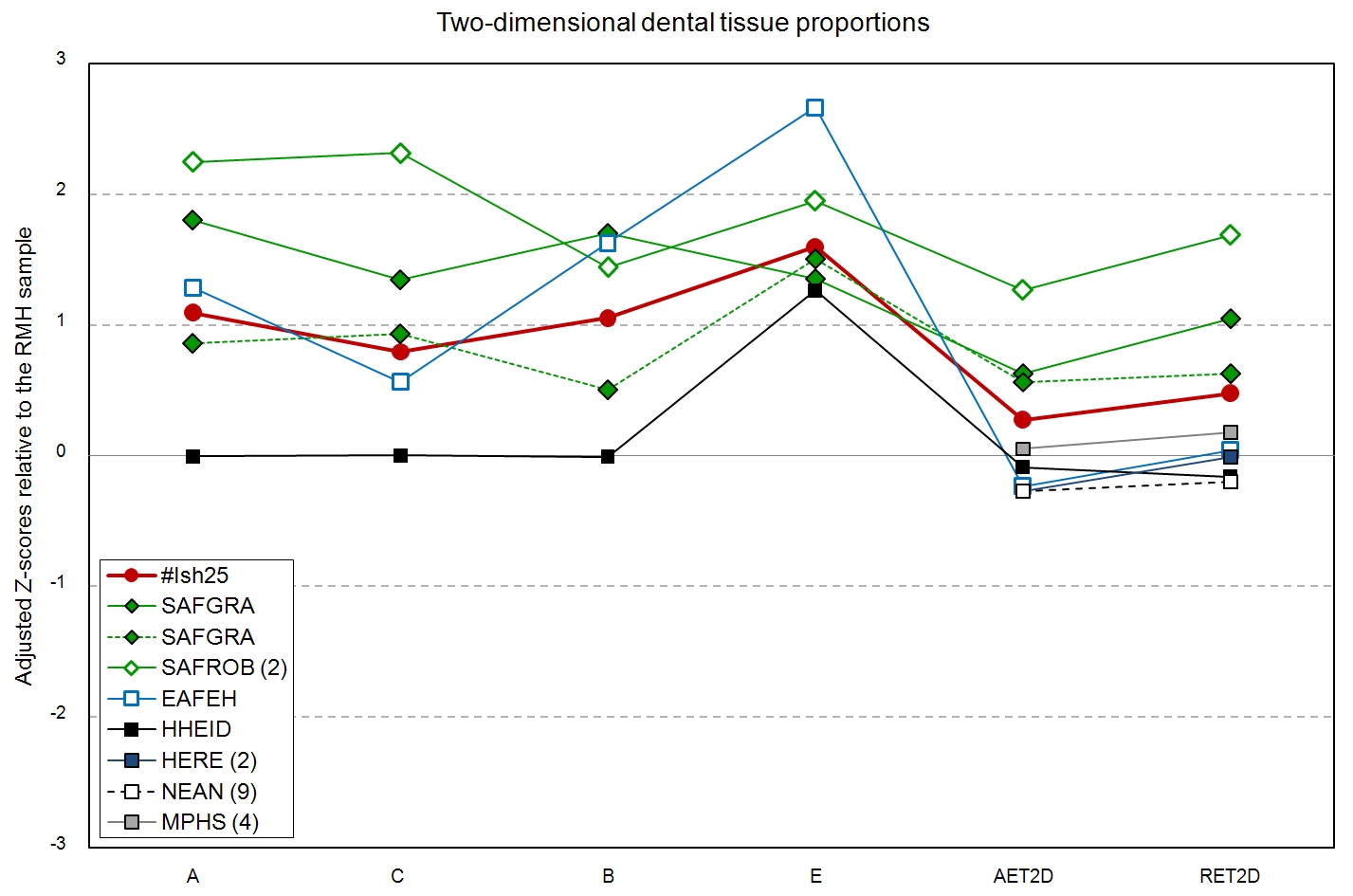
**

**Figure S13.** Adjusted Z-scores of the two-dimensional dental tissue proportions of #Ish25.

It is compared to the mean and the range of variation of pooled recent modern human samples. Adjusted Z-scores of specimens or average values of comparative groups of australopiths and fossil *Homo* are also displayed. The number of specimens for the fossil comparative groups is given between brackets. The zero-line represents the mean of the RMH sample, the -1 / + 1 lines represents the 95% lower and upper limits of the RMH variation. Comparative samples are defined in Table S2, and the measurements in Table S3.

**
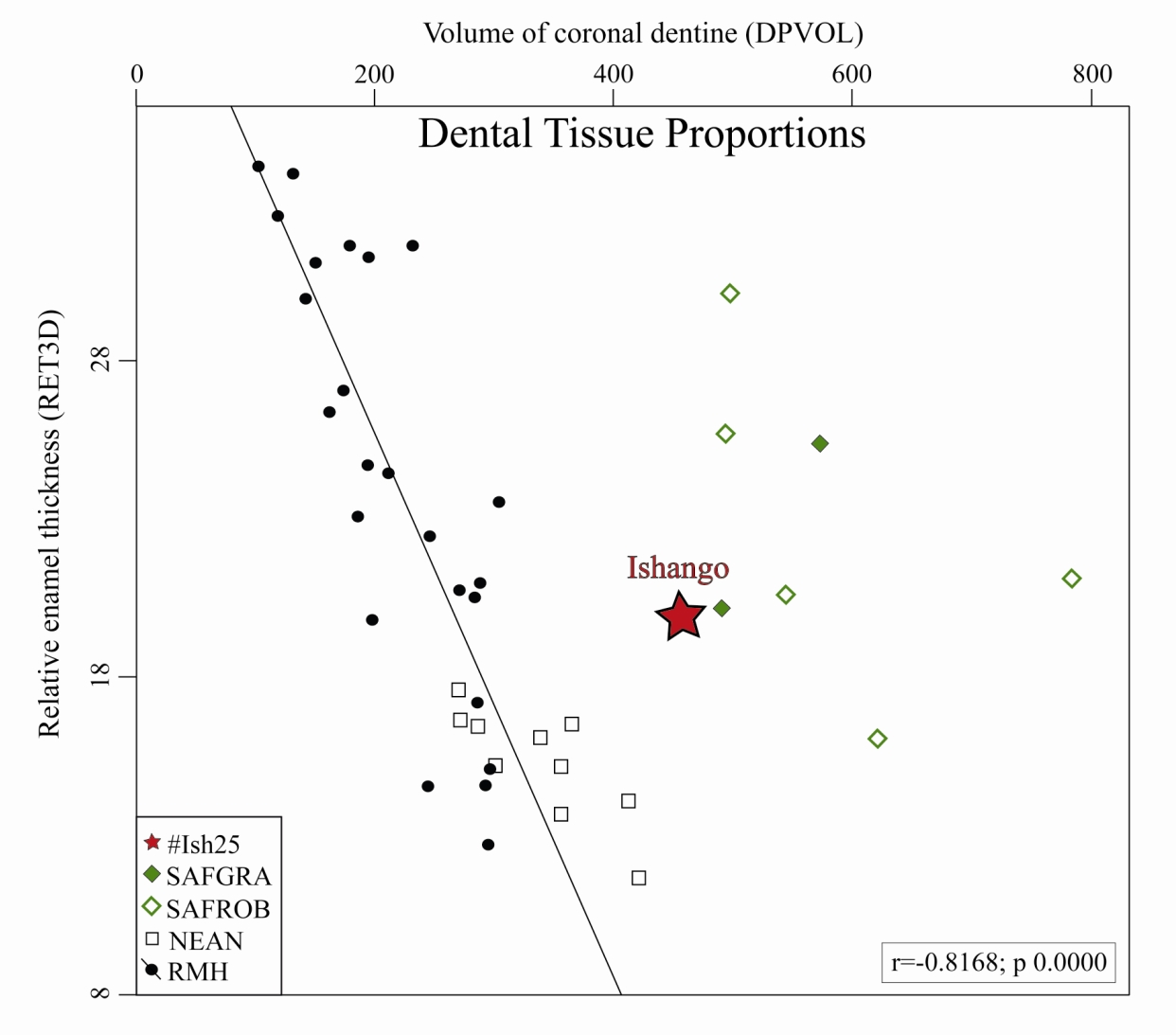

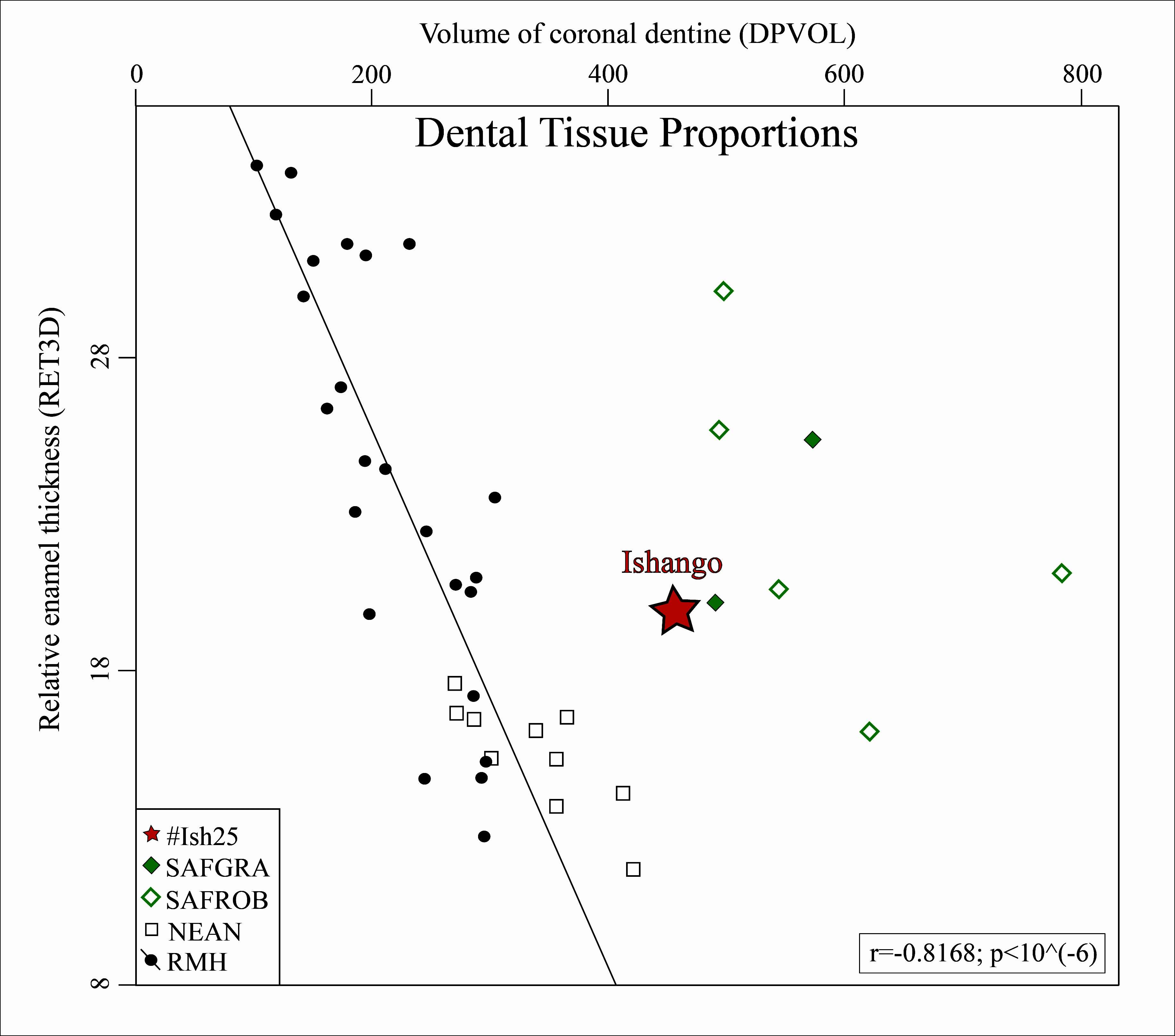
**

**Figure S14.** Bivariate plot of the volume of coronal dentine (DPVOL) and the relative enamel thickness (RET3D).

The regression line for the recent modern human sample is illustrated. Comparative samples as in Table S2.

Text S4.4 Enamel-dentine junction (EDJ) morphology

Since, it has been demonstrated that the shape of the EDJ reliably discriminates between closely related species of extant apes [138], as well as of fossil hominins [139], landmarks and semilandmarks were used to characterize and compare the EDJ morphology of the Ishango molar. This analysis involved the following steps: microtomographic scanning and EDJ surface generation, geometric morphometric analysis, principal components and canonical variates analysis, and visualization of shape differences between #Ish25 and comparative taxa. Table S5 lists the first molar comparative sample and Table S6 lists the second molar comparative sample. The latter was only used to confirm that #Ish25 is a first molar (see below).

*Microtomography and EDJ surface generation*

Each molar was microCT scanned at the Max Planck Institute for Evolutionary Anthropology in Leipzig, Germany using either an industrial or desktop microCT system with a resultant voxel resolution ranging from 14 – 70µm. The image stacks of each tooth were filtered (using a computer programmed macro that employs a three-dimensional median and mean-of-least-variance filter) to improve tissue grayscale homogeneity and then manually segmented into enamel and dentine components using Avizo (v6.1). The EDJ of each molar was extracted as a digital surface model (.ply format).

*Geometric morphometric analysis*

The EDJ surface models were imported into Avizo for the collection of three sets of 3D anatomical landmarks (Figure S15). The first set (referred to as ‘MAIN’) included four landmarks: one on the tip of the dentine horn of each primary cusp. The second set includes 50 to 70 landmarks along the tops of the ridges that connect the four dentine horns. This set of points forms a continuous line, beginning at the tip of the protocone and moving in a buccal direction. The third set includes about 40 landmarks along the cervix of the molar, beginning at the midpoint of the buccal face and continuing in a lingual direction. As the ridge and cervix curves were later resampled (see below), it was not initially necessary that the same number of points be placed along the curve for each specimen. Thus, the spacing of points was dictated such that they did not touch adjacent neighbours, but was not so far apart as to misrepresent aspects of the curve (as represented in Figure S15).

The process by which a corresponding set of landmarks and semilandmarks [140-141] was generated for each specimen was as follows. First, for the ridge and cervix landmarks a smooth curve was fitted by starting at an initial point (the first collected landmark) and moving lingually around the curve. A cubic spline was used so that the curve is forced to pass through each measured coordinate. The four MAIN homologous landmarks were projected onto the ridge curve, dividing it into four sections. For each section, a large sample of very closely spaced coordinates was computed along the curve, and the distances between adjacent coordinates were calculated and summed together to approximate the length along the curve segments between the MAIN landmarks. Each length was divided by a given number based on an estimate of the relative contribution of each section of the ridge curve across the molars in the study sample (protocone-paracone = 18; paracone-metacone = 15; metacone-hypocone = 12; hypocone-protocone = 15), and the coordinate location at each equally spaced distance was recorded (Figure S15). This step was repeated for the cervix curve, but in this case 30 equally spaced landmarks were derived. Thus, at this stage, all specimens had the same total number of landmarks (*i.e.* the homologous, fixed, landmarks on the tips of the dentine horns, plus equal numbers of semilandmarks on the ridge and cervix curves). We used the algorithm described by Gunz *et al.* [141-142], which allows semilandmarks to slide along tangents to the curve. These tangents were approximated for each semilandmark as the vector between the two neighbouring points. Semilandmarks were iteratively allowed to slide along their respective curves to minimize the bending energy of the thinplate spline interpolation function computed between each specimen and the Procrustes average for the sample. After the application of the sliding algorithm, the four fixed landmarks and the 90 semilandmarks for the curves were considered homologous among each of the study specimens.

Each set of landmarks and semilandmarks was converted to shape coordinates by generalized least squares Procrustes superimposition [143-144]. This removed information about location and orientation from the raw coordinates and standardized each specimen to unit centroid size; a size-measure computed as the square root of the sum of squared Euclidean distances from each semilandmark to the specimen’s centroid [145]. All data processing was done in Mathematica v6.0 (www. wolfram.com) using a software routine written by Philipp Gunz.


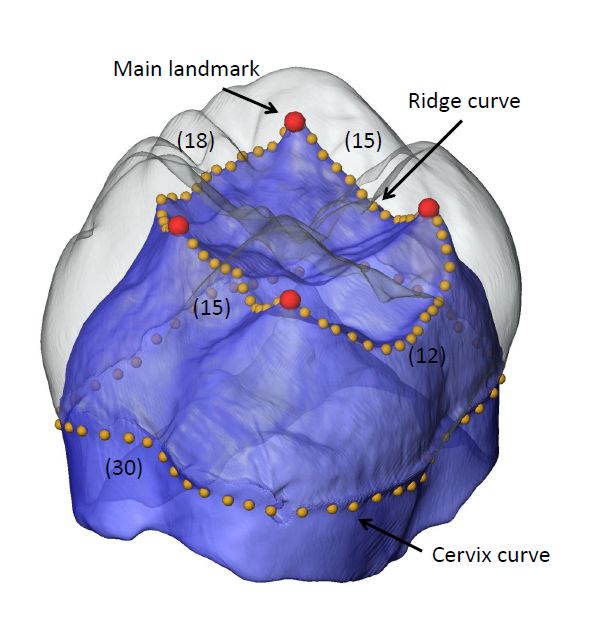


**Figure S15.** Illustration of landmarks collected on the ridge curve, cervix curve and main dentine horns.

Numbers in brackets represent the number of resampled landmarks for each curve (see text for details). The enamel cap has been made transparent.

Table S5: First molar sample used to analyze EDJ shape of the #Ish25 molar.

| Group* | N | Specimens |
| --- | --- | --- |
| SAFGRA | 10 | Sterkfontein: Sts 1, 8, 24, 56, 57, Stw 183, 252J, 402, 450  Taung: Taung 1 |
| SAFROB | 13 | DNH 57B, 60A  Swartkrans: SK 13.14, 16.1591, 47, 48, 52, 102, 829, 832, 838a, 89a  Kromdraai: TM 1517 |
| EH | 5 | Drimolen: DNH 39, 70  Koobi Fora: KNM-ER 1590L  Swartkrans: SKX 268  Sterkfontein: STW 151 |
| HER | 2 | Sangiran: S4, S7 3b |
| NEAN | 14 | Krapina: KPR 45, 46, D100, D134, D136, D171  La Quina: H18  Kebara: KMH 21  Combe-Grenal: XIII  Bourgeois-Delaunay: BD8  Roc de Marsal: Roc de Marsal 1  Scladina : 4A  El Sidron : SD 1105  Steinheim : Steinheim 1 |
| MPHS | 5 | Qafzeh: Qafzeh 9, 10, 11, 15  Skhul: Skhul 1 |
| RMH | 19 | National Museum of Natural History: 10 molars of unknown provenience  MPI-EVA: 5 molars from clinical extractions from Germany  Royal Belgian Institute of Natural Sciences RBINS, Belgium : 4 Neolithic specimens |
| Total | 68 |  |

*SAFGRA: *Australopithecus africanus*; SAFROB: *Paranthropus robustus*; EH: Early *Homo*; HER: *Homo erectus*; NEAN: Specimens attributed to Neandertal lineage from Eurasia; MPHS: Middle Palaeolithic *Homo sapiens* from Southwest Asia; RMH: Recent modern humans. N: number.

*Principal components and canonical variates analysis*

To assess the taxonomic affinity of the Ishango molar, based on EDJ shape, we performed a principal component analysis in shape space on the Procrustes coordinates of each specimen. We then assessed the classification of #Ish25 as a first or second molar using a cross-validated canonical variates analysis using 5-20 PCs. Table S7 summarizes the classification accuracy of the comparative sample. Classifications to tooth position (92-100%), taxon (92-100%) and tooth position AND taxon (83-100%) are very high and suggest that EDJ morphology can be used to determine the tooth position and taxonomic affinity of #Ish25.

When classified in the combined M1/M2 comparative sample #Ish25 classifies as follows (see Table S5 for group abbreviations): 47% MPHS first molar, 23% MPHS second molar, 18% EH first molar, 11% SAFGRA first molar, and 11% SAFROB first molar. Regarding tooth position this suggests that #Ish25 is a first molar, which is consistent with other qualitative aspects of tooth crown morphology (see main text).

Treating #Ish25 as a first molar, we then assessed its taxonomic affiliation using a cross-validated canonical variates analysis of the principal components of shape variation (using each of 5-20 PCs). In this case #Ish25 classifies 68% to a combined EH/ERECT group and 32% as MPHS or, if we exclude the two Sangiran specimens 58% to EH, 31% to MPHS and 11% to SAFROB.

Table S6: Second molar sample used to assess the classification of #Ish25 as a first molar.

| Group* | N | Specimens |
| --- | --- | --- |
| SAFGRA | 12 | Sterkfontein: Sts 8, 22, 28, 30, 52a, 56, Stw 183, 188, 252I, 280, 498a, TM 1511 |
| SAFROB | 10 | Swartkrans: SK13.14, 16.1591, 47, 48, 49, 52, 826a2, 834, SKW 33,  Kromdraai: TM 1517 |
| EH | 5 | Koobi Fora: KNM-ER 1590, 1808H, 1813  Swartkrans: SK 27  Sterkfontein: SE 1508 |
| NEAN | 18 | Krapina: D96, D98, D101, D135, D165, D166, D169, D175, D177  Combe-Grenal: IX  Bourgeois-Delaunay: BD8  Le Moustier: Le Moustier  Scladina: 4A  El Sidron: SD 4, 407, 551  Saint-Césaire: Saint-Césaire 1  Steinheim: Steinheim 1 |
| MPHS | 7 | Qafzeh: 9, 10, 11, 15  Equus Cave: EQ H10  Combe Capelle: Combe Capelle  Oberkassel: D999 |
| RMH | 22 | National Museum of Natural History: 1 molar of unknown provenience  MPI-EVA: 9 molars from clinical extractions from Germany, 8 archeological specimens  Royal Belgian Institute of Natural Sciences RBINS, Belgium : 4 Neolithic specimens |
| Total | 74 |  |

*Abbreviation as in Table S5.

Table S7. Classification of the M1/M2 comparative sample using a cross-validated CVA.

| Taxon | N | Tooth position | Taxon | Tooth and Taxon |
| --- | --- | --- | --- | --- |
| RMH | 41 | 98% | 95% | 93% |
| MPHS | 12 | 92% | 92% | 83% |
| NEAN | 32 | 94% | 100% | 94% |
| EH | 10 | 100% | 100% | 100% |
| SAFGRA | 23 | 100% | 95% | 95% |
| SAFROB | 23 | 100% | 96% | 96% |

*Abbreviation as in Table S5. Note that the HER specimens from Sangiran were not included in this assessment of overall classification accuracy.

*Visualization of #Ish25 EDJ shape relative to comparative taxa*

By calculating the mean shape of the first molar EDJ ridge and cervix curves for Plio-Pleistocene (including SAFGRA, SAFROB, EH) and Upper Pleistocene (including NEAN, MPHS, RMH), we are able to visualize the shape similarity of #Ish25 to these two groups. Figure S16 illustrates the fact that in both shape and form space #Ish25 more closely resembles the EDJ of the Plio-Pleistocene sample.

**
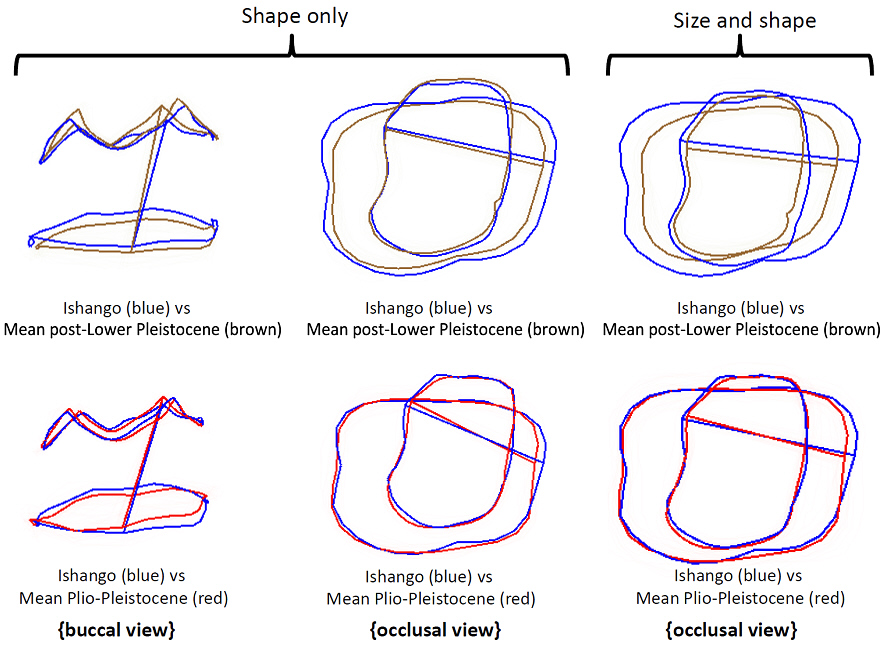
**

**Figure S16.** Comparison of the EDJ shape of #Ish25.

The blue lines represent the measured EDJ ridge and cervix curves of #Ish25. The mean shape of the post-Lower Pleistocene sample is given in brown (top), and the mean shape of the Plio-Pleistocene sample in red (bottom).

**References**

1. de Heinzelin J, Verniers J (1996) Realm of the Upper Semliki (Eastern Zaire). An essay on historial geology. Tervuren: Royal Museum of Central Africa. 87 p.

2. de Heinzelin J (1961) Le Paléolithique aux abords d'Ishango. Bruxelles: Institut des Parcs nationaux du Congo et du Ruanda-Urundi. 34 p.

3. Brooks AS, Helgren D, Cramer JS, Franklin A, Hornyak W, et al. (1995) Dating and Context of Three Middle Stone Age Sites with Bone Points in the Upper Semliki Valley, Zaire. Science 268: 548-553.

4. Pickford M, Senut B, Hadoto D (1993) Geology and Palaeobiology of the Albertine Rift Valley, Uganda-Zaire. Volume I: Geology. Orléans: C.I.F.E.G. 190 p.

5. Senut S, Pickford M, Ssemmanda I, Elepu D, Obwona P (1987) Découverte du premier Homininae (*Homo sp.*) dans le Pléistocène de Nyabusosi (Ouganda Occidental). C R Acad Sci Paris 305: 819-822.

6. Pickford M, Senut B, Roche H, Mein P, Ndaati G, et al. (1989) Uganda Palaeontology Expedition : résultats de la deuxième mission (1987) dans la région de Kisegi-Nyabusosi (bassin du lac Albert, Ouganda). C R Acad Sci Paris 308: 1751-1758.

7. Todd NE (2005) Reanalysis of African *Elephas recki*: implications for time, space and taxonomy. Quatern Int 126-128: 65-72.

8. Kalb JE, Jolly CJ, Tebedge S, Mebrate A, Smart C, et al. (1982) Vertebrate faunas from the Awash Group, Middle Awash Valley, Afar, Ethiopia. J Vertebr Paleontol 2: 237-258.

9. Clark JD, de Heinzelin J, Schick KD, Hart WK, White TD, et al. (1994) African *Homo erectus*: old radiometric ages and young Oldowan assemblages in the Middle Awash Valley, Ethiopia. Science 264: 1907-1910.

10. McBrearty S (1999) The archaeology of the Kapthurin Formation. In Andrews P, Banham P, editors. Late Cenozoic environments and Hominid Evolution: a tribute to Bill Bishop. London: Geological Society of London. pp. 143-156.

11.Alemseged Z, Geraads D (2000) A new Middle Pleistocene fauna from the Busidima–Telalak region of the Afar, Ethiopia. C R Acad Sci Paris 331: 549-556.

12. Pickford M, Senut B, Poupeau G, Brown F, Haileab B (1991) Correlation of tephra layers from the Western Rift Valley (Uganda) to the Turkana Basin Geology (Ethiopia/Kenya) and the Gulf of Aden. C R Acad Sci Paris 313: 223-229.

13. Harris JWK, Williamson PG, Morris PJ, de Heinzelin J, Verniers J, et al. (1990) Archaeology of the Lusso Beds. In: Boaz NT, editor. Evolution of Environments and Hominidae in the African Western Rift Valley. Martinsville: Virginia Museum of Natural History. pp. 237-272.

14. Harris JWK, Williamson PG, Verniers J, Tappen MJ, Stewart K, et al. (1987) Late Pliocene hominid occupation in Central Africa: the setting, context, and character of the Senga 5A site, Zaire. J Hum Evol 16: 701-728.

15. de Heinzelin J, Verniers J (1996) Realm of the Upper Semliki (Eastern Zaire). An essay on historial geology. Tervuren: Royal Museum of Central Africa. 87 p.

16. Verniers J, de Heinzelin J (1990) Stratigraphy and Geological History of the Upper Semliki: A preliminary Report. In: Boaz NT, editor. Evolution of Environments and Hominidae in the African Western Rift Valley. Martinsville: Virginia Museum of Natural History. pp. 17-39.

17. Damas H (1940) Observations sur les couches fossilifères bordant la Semliki. Revue de Zoologie et de Botanique Africaines 33: 265-272.

18. de Heinzelin J (1957) Les fouilles d'Ishango. Bruxelles: Institut des Parcs nationaux du Congo belge. 128 p.

19. de Heinzelin J (1955) Le fossé tectonique sous le parallèle d'Ishango. Bruxelles: Institut des Parcs nationaux du Congo belge. 150 p.

20. Boaz NT (1990) The Semliki Research Expedition: History of Investigation, Results, and Background to Interpretation. In: Boaz NT, editor. Evolution of Environments and Hominidae in the African Western Rift Valley. Martinsville: Virginia Museum of Natural History. pp. 3-14.

21. Brooks AS, Smith CC (1987) Ishango revisited: new age determinations and cultural interpretations. Afr Archaeol Rev 5: 65-78.

22. Helgren DM (1977) Locations and Landscapes of Paleolithic Sites in the Semliki Rift, Zaire. Geoarchaeology 12: 337-361.

23. Twiesselmann F (1958) Exploration du Parc National Albert. Mission J. de Heinzelin de Braucourt (1950). Les ossements humains du gîte mésolithique d'Ishango. Bruxelles: Institut des Parcs nationaux du Congo belge. 125 p.

24. Greenwood PH (1959) Quaternary fish-fossils. Bruxelles: Institut des Parcs nationaux du Congo belge. 120 p.

25. Hopwood AT, Misonne X (1959) Mammifères fossiles. Bruxelles: Institut des Parcs nationaux du Congo belge. 120 p.

26. Verheyen W (1959) Oiseaux fossiles. Bruxelles: Institut des Parcs nationaux du Congo belge. 120 p.

27. Stewart KM (1989) Fishing Sites of North and East Africa in the Late Pleistocene and Holocene. Environmental Change and Human Adaptation. Oxford: BAR international series. 273 p.

28. Peters J (1990) Late Pleistocene hunter-gatherers at Ishango (Eastern Zaire): The faunal evidence. Revue de Paléobiologie 9: 73-112.

29. Mercader J, Brooks AS (2001) Across Forests and Savannas: Later Stone Age Assemblages from Ituri and Semliki, Democratic Republic of Congo. J Anthropol Res 57: 197-217.

30. Brooks AS, Robertshaw P (1990) The Glacial Maximum in tropical Africa: 22 000-12 000 BP. In: Gamble C, Soffer O, editors. The World at 18 000 BP: Low Latitudes. Volume 2. London: Unwin Hyman. pp. 121-169.

31. Fuchs V (1934) The geological work of the Cambridge expedition to the East African lakes, 1930-1931. Geological magazine 71:97-112; 72:145-166.

32. Adam W (1957) Mollusques quaternaires de la région du lac Edouard. Bruxelles: Institut des Parcs nationaux du Congo belge. 172 p.

33. Van Damme D, Pickford M (1994) The Late Cenozoic freshwater molluscs of the Albertine Rift, Uganda-Zaire: Evolutionary and Palaeoecological implications. In: Senut S, Pickford M, editors. Geology and palaeobiology of the Albertine Rift Valley, Uganda-Zaire. Volume 2, Palaeobiology. Orléans: C.I.F.E.G. pp. 71-87.

34. Van Neer W (1994) Cenozoic fish fossils from the Albertine Rift Valley in Uganda. In: Senut S, Pickford M, editors. Geology and palaeobiology of the Albertine Rift Valley, Uganda-Zaire. Volume 2, Palaeobiology. Orléans: C.I.F.E.G. pp. 89-128.

35. Brown FH, McDougall I, Davies T, Maier R (1985) An Integrated Plio-Pleistocene Chronolgy for the Turkana Basin. In: Delson E, editor. Ancestors: The Hard Evidence. New York: Alan R. Liss. pp. 82-90.

36. Cooke HBS (1990) Suid Remains from the Upper Semliki Area, Zaire. In: Boaz NT, editor. Evolution of Environments and Hominidae in the African Western Rift Valley. Martinsville: Virginia Museum of Natural History. pp. 197-201.

37. Boaz NT, Bernor RL, Brooks AS, Cooke HBS, de Heinzelin J, et al. (1992) A new evaluation of the significance of the Late Neogene Lusso Beds, Upper Semliki Valley, Zaire. J Hum Evol 22: 505-517.

38. Kirchner MT, Edwards HGM, Lucy D, Pollard AM (1997) Ancient and Modern Specimens of Human Teeth: a Fourier Transform Raman Spectroscopic Study. J Raman Spectrosc 28: 171-178.

39. Dauphin Y, Williams CT (2007) The chemical compositions of dentine and enamel from recent reptile and mammal teeth-variability in the diagenetic changes of fossil teeth. CrystEngComm 9: 1252-1261.

40. Person A, Bocheren H, Saliège J-F, Paris F, Zeitoun V, et al. (1995) Early Diagenetic Evolution of Bone Phosphate: An X-ray Diffractometry Analysis. J Archaeol Sci 22: 211–221.

41. Person A, Bocherens H, Mariotti A, Renard M (1996) Diagenetic evolution and experimental heating of bone phosphate. Palaeogeogr. Palaeoclimatol. Palaeoecol. 126: 135-149.

42. Bertoluzza A, Brasili P, Castri L, Facchini F, Fagnano, et al. (1997) Preliminary Results in Dating Human Skeletal Remains by Raman Spectroscopy. J Raman Spectrosc 28: 185-188.

43. Tsuda H, Ruben J, Arends J (1996) Raman spectra of human dentin mineral. Eur J Oral Sci 104: 123-131.

44. Orban R, Semal P, Twiesselmann F (2001) Sur la biométrie des mandibules et des dents humaines d'Ishango (LSA, République Démocratique du Congo). Bull Mém Soc Anthropol Paris 13: 97-109.

45. Korenhof CAW (1960) Morphogenetical aspects of the human upper molar: a comparative study of its enamel and dentine surfaces and their relationship to the crown pattern of fossil and recent primates. Utrecht: Uitgeversmaatschappij Neerlandia. 368 p.

46. Wood BA, Engleman CA (1988) Analysis of the dental morphology of Plio-Pleistocene hominids. V. Maxillary postcanine tooth morphology. J Anat 161: 1-35.

47. Robinson JT (1956) The dentition of the Australopithecinae. Pretoria: Transvall Museum. 179 p.

48. Clarke RJA (2012) *Homo habilis* maxilla and other newly-discovered hominid fossils from Olduvai Gorge, Tanzania. J Hum Evol 63: 418-428.

49. Wood B (1991) Koobi Fora Research Project, Volume 4, Hominid Cranial Remains. Oxford: Oxford University Press. 466 p.

50. Abbazi L, Fanfani F, Ferretti MP, Rook L (2000) New Human Remains of Archaic *Homo sapiens* and Lower Palaeolithic Industries from Visogliano (Duino Aurisina, Trieste, Italy) J Archaeol Sci 27: 1173-1186.

51. Bartucz L (1940) Der Urmensch der Mussolini Hohle. Bei cserepfalu Geol Hungarica ser Paleont f 14: 47-105.

52. Bay R (1981) Der menschliche Oberkiefer aus der Grotte de Cotencher (Rochefort, Neuchâtel, Suisse). Arch Suisses d'Anthrop Gén 54: 57-101.

53. Bay R (1984) The human maxilla from grotte de Contencher (Rochefort, Neuchâtel, Switzerland). J Hum Evol 13: 301-305.

54. Billy G (1975) Etude anthropologique des restes humains de l'abri Pataud. In Movius HL, editor. Excavation of the Abri Pataud, Les Eyzies (Dordogne). Cambridge: Peabody Museum of Archaeology and Ethnology, Harvard University press. pp. 201-261.

# 55. Bräuer G, Melhman MJ (1988) **Hominid molars from a middle stone age level at the Mumba Rock Shelter, Tanzania. Am J Phys Anthropol 75: 69-76.**

56. Brink JS, Herries AIR, Moggi-Cecchi J, Gowlett JAJ, Bousman B, et al. (2012) First hominine remains from a ~1.0 million year old bone bed at Cornelia-Uitzoek, Free State Province, South Africa. J Hum Evol 63: 527–535.

57. Bonin Von G (1935) The Magdalenian Skeleton from Cap-Blanc in the Field Museum of Natural History. Urbana: University of Illinois. 76 p.

58. Brown F, Harris J, Leakey R, Walker A (1985) Early *Homo erectus* skeleton from west Lake Turkana, Kenya. Nature 316: 788-792.

59. Brown B, Walker A (1993) The dentition. In Walker A, Leakey R, editors. The Nariokotome *Homo erectus* skeleton. Cambridge: Harvard University Press. p 161-192.

60. Cattani L, Cremaschi M, Ferraris MR, Mallegni F, Masini F, et al. (1991) Les gisements du Pléistocène moyen de Visogliano (Trieste): restes humains, industries, environnement. L'Anthropologie 95: 9-36.

61. Chamla MC (1980) Etude des variations métriques des couronnes dentaires des Nord-Africains, de l'Epipaléolithique à l'époque actuelle. L'Anthropologie 84: 254-271.

62. Condemi S (1992) Les Hommes fossiles de Saccopastore et leurs relations phylogénétiques. Paris: Editions du CNRS. 174 p.

63. Condemi S (2001) Les Néandertaliens de La Chaise. Paris: Editions du CTHS. 178 p.

64. Crevecoeur I (2008) Etude anthropologique du squelette du Paléolithique supérieur de Nazlet Khater 2 (Egypte). Apport à la compréhension de la variabilité passée des hommes modernes. Leuven: Leuven University Press. 318 p.

65. Defleur A, Dutour O, Vandermeersch B (1992) Etude de deux dents humaines provenant des niveaux moustériens de La Baume Néron (Soyons, Ardèche). Bull Mem Soc Anthropol Paris 4: 127-134.

66. Frayer DW (1978) The evolution of the dentition in Upper Palaeolithic and Mesolithic Europe. Laurens: University of Kansas. 201 p.

67. Frayer DW, Jelínek J, Oliva M, Wolpoff MH (2006) Aurignacian male crania jaws and teeth from Mladec caves. In Teschler-Nicola M, editor. Early modern humans at the Moravian gate: the Mladeč caves and their remains. Wien: Springer. pp. 185-272.

68. Gambier D (1986) Etude préliminaire des crânes magdaléniens de la grotte de Montgaudier (Charente). Bull Mem Soc Archéol et Hist Charente 1: 31-35.

69. Gambier D, Lenoir M (1991) Les vestiges humains du Paléolithique supérieur en Gironde. Bull Soc Anthropol Sud-Ouest 26: 1-31.

70. Garralda MD, Vandermeersch B (2000) Les Néandertaliens de la grotte de Combe-Grenal (Domme, France). Paléo 12: 213-259.

71. Genet-Varcin E (1974) Etude de dents humaines isolées provenant des grottes de La Chaise de Vouthon (Charente). Bull Mem Soc Anthropol Paris 1: 373-381.

72. Genet-Varcin E (1982) Vestiges humains du Würmien inférieur de Combe-Grenal, commune de Domme (Dordogne)*.* Ann Paleontol 68: 133-169.

73. Graziosi P (1947) Gli uomini paleolitici della grotta di S. Teodoro (Messina). Riv Sci Biol 2:125-208.

74. Green HS, Stringer CB, Collcutt SN, Currant AP, Huxtabe, et al. (1981) Pontnewydd Cave in Wales - a new Middle Pleistocene hominid site. Nature 294: 707-713.

75. Gremyatskij MA, Nesturkh MF (1949) Teshik-Tash. Moscow: Moscow State University. 183 p.

76. Grine FE (1989) New Hominid Fossils From the Swartkrans Formation (1979-1986 Excavations): Craniodental Specimens. Am J Phys Anthropol 79: 409-449.

77. Heim JL (1976) Les hommes fossiles de La Ferrassie. 1. Le Gisement, les squelettes adultes, crâne et squelette du tronc. Paris: Masson. 331 p.

78. Heim JL (1991) L'enfant magdalénien de la Madeleine. L'Anthropologie 95: 611-638.

79. Hillson SW (2006) Dental morphology. proportions and attrition. In Trinkaus E, Svoboda J, editors. Early modern Human Evolution in Central Europe. The People of Dolní Vĕstonice and Pavlov. Oxford: Oxford University Press. p. 179-223.

80. Keith A, Knowles FHS (1911) A Description of Teeth of Palaeolithic Man from Jersey. J Anat Physiol 46: 12-27.

81. Keyser AW (2000) The Drimolen skull: the most complete australopithecine cranium and mandible to date. S Afr J Sci 96: 189-192.

82. Kieser JA (1990) Human adult odontometric: the study of variation in adult tooth size. Cambridge: Cambridge University Press. 208 p.

83. Kimbel WH, Johanson D, Rak Y (1994) The first skull and other new discoveries of *Australopithecus afarensis* at Hadar, Ethiopia. Nature 368: 449-451.

84. Kimbel WH, Johanson D, Rak Y (1997) Systematic Assessment of a Maxilla of *Homo* From Hadar, Ethiopia. Am J Phys Anthropol 103: 235-262.

85. Klaatsch H (1909) Preuves que l'*Homo mousteriensis hauseri* appartient au type du Néandertal. L'homme Préhistorique 7: 10-16.

86. Leakey MG, Spoor F, Dean C, Feibel CS, Anton SC et al. (2012) New fossils from Koobi Fora in northern Kenya confirm taxonomic diversity in early *Homo*. Nature 488: 201-204.

87. Leroi-Gourhan A (1958) Etude des restes humains fossiles provenant des grottes d'Arcy-sur-Cure. Ann Paleontol 44: 87-148.

88. Lumley de MA (1973) Anténéandertaliens et néandertaliens du bassin méditerranéen occidental européen. Etudes quaternaires. Marseille: Université de Provence. 625 p.

89. Magori CC, Day MH (1983) Laetoli Hominid 18: an early *Homo sapiens* skull. J Hum Evol 12: 747-753.

90. Mallegni F (1992) Squelette de femme d’une sépulture des couches gravettiennes de la Grotta Paglicci près de Rignano Garganico (Pouilles, Italie): Paglicci 25. Riv Antropol 30: 209-216.

91. Mallegni F, Parenti R (1972-1973) Studio antropologico di uno scheletro giovanile d’epoca gravettiana raccolto nella grotta Paglicci (Rignano Garganico). Riv Antropol 58: 317-342.

92. Martin H (1923) Recherches sur l'évolution du Moustérien dans le gisement de La Quina (Charente). 3e vol., L'homme-fossile*.* Angoulême: Imprimerie ouvrière. 260 p.

93. Matiegka J (1934) *Homo predmostensis*, fosilni clovek z Predmosti na Morave. 1, Lebky. Praha: Nakladem ceske akademie ved a umeni. 145 p.

94. McCown TD, Keith A (1939) The Stone age of Mount Carmel. The fossil human remains from the Levalloiso-mousterian. vol. II. Oxford: Clarenton press. 390 p.

95. Ménard J (2002) Etude odontologique des restes atériens de Dar-es-Soltan II et d'El-Harhoura II au Maroc. Bull Archéologie Marocaine 19: 67-118.

96. Moggi-Cecchi J, Grine FE, Tobias P (2006) Early hominid dental remains from Members 4 and 5 of the Sterkfontein Formation (1966-1996 excavations): Catalogue, individual associations, morphological descriptions and initial metrical analysis. J hum Evol 50: 239-328.

97. Moggi-Cecchi J, Menter C, Boccone S, Keyser A (2010) Early hominin dental remains from the Plio-Pleistocene site of Drimolen, South Africa. J hum Evol 58: 374-405.

98. Paoli G, Parenti R, Sergi S (1980) Gli scheletri mesolitici della Caverna delle Arene Candide (Liguria). Memorie dell'Istituto Italiano di Paleontologia Umana Roma 3: 33-154.

99. Pap I, Tillier AM, Arensburg B, Chech M (1996) The Subalyuk Neanderthal remains (Hungary): a re-examination. Annales historico-naturales Musei nationalis hungarici 88: 233-270.

100. Pardini E, Lombardi Pardini EC (1981) I Paleolitici di Vado all'Arancio (Grosseto). Archivio per l'Antropologia e la Etnologia Firenze 111: 75-119.

101. Patte E (1962) La dentition des Néandertaliens. Paris: Masson et Cie. 307 p.

102. Prat S, Brugal JP, Roche H, Texier PJ (2003) Nouvelles découvertes de dents d’hominidés dans le membre Kaitio de la Formation de Nachukui (1,65–1,9 Ma), Ouest du lac Turkana (Kenya). C R Palevol 2: 685–693.

103. Protsch R (1981) The palaeoanthropological finds of the Pliocene and Pleistocene. Tübingen Monographien zur Urgeschiscte Band 4.3: 31-181.

104. Radovčić J, Smith FH, Trinkaus E, Wolpoff M (1988) The Krapina Hominids: An Illustrated Catalog of Skeletal Collection. Zagreb: Mladost. 118 p.

105. Riquet R (1973) Le crâne magdalénien des Hoteaux (Ain). Bull Soc Anthropol Sud-Ouest 9: 15-23.

106. Siffre A (1908) Etude des dents humaines du Petit-Puymoyen (Charente). Revue de l'Ecole d'Anthropologie de Paris 18: 66-72.

107. Sládek V, Trinkaus E, Hillson SW, Holliday TW (2000) The People of the Pavlovian: Skeletal Catalogue and Osteometrics of the Gravettian Fossil Hominids from Dolní Vĕstonice and Pavlov (Brno: Akademie vĕd Česke republicky. 244 p.

108. Soficaru A, Doboş A, Trinkaus E (2006) Early modern humans from the Peştera Muierii, Baia de Fier, Romania. P Natl Acad Sci USA 103: 17196-17121.

109. Suwa G, Asfaw B, Haile-Selassie Y, White T, Katoh S, et al (2007) Early Pleistocene *Homo erectus* fossils from Konso, southern Ethiopia. Anthropol Sci 115: 133-151.

110. Suzuki H, Takai F (1970) The Amud Man and his cave site. Tokyo: The University of Tokyo. 64 p.

111. Tillier AM (1979) La dentition de l'enfant moustérien Chateauneuf 2 découvert à l'abri de Hauteroche (Charente). L'Anthropologie 83: 417-438.

112. Tillier AM (1999) Les enfants moustériens de Qafzeh. Paris: Editions CNRS. 239 p.

113. Trinkaus E (1983) The Shanidar Neandertals. New York: Academic press. 502 p.

114. Trinkaus E, Milota S, Rodrigo R, Mircea G, Moldovan O (2003) Early modern human cranial remains from the Pestera cu Oase, Romania. J hum Evol 45: 245-253.

115. Vallois HV (1952) Les restes humains du gisement moustérien de Monsempron. Ann Paleontol (Vert) 38: 100-120.

116. Vallois HV (1958) La grotte de Fontéchevade. Deuxième partie. Anthropologie. Paris: Masson. 263 p.

117. Vandermeersch B (1981) Les Hommes fossiles de Qafzeh (Israël). Paris: Editions du CNRS. 319 p.

118. Verna C (2006) Les restes humains moustériens de la station amont de La Quina (Charente. France). PhD University of Bordeaux 1. 629 p.

119. Vigier D (1984) Contribution à l'étude de restes humains aziliens. PhD University of Bordeaux 2. 52 p.

120. Ward CV, Leakey MG, Walker A (2001) Morphology of *Australopithecus anamensis* from Kanapoi and Allia Bay, Kenya. J Hum Evol 41: 255-368.

121. White TD, Asfaw B, DeGusta D, Gilbert H, Richard GD, et al. (2003) Pleistocene *Homo sapiens* from Middle Awash, Ethiopia. Nature 423: 742-747.

122. Wolpoff MH (1979) The Krapina dental remains. Am J Phys Anthropol 50: 67-114.

123. Scolan H, Santos F, Tillier AM, Maureille B, Quintard A (2012) Des nouveaux vestiges néanderthaliens à Las Pélénos (Monsempron-Libos, Lot-et-Garonne, France). Bull Mem Soc Anthropol Paris 24: 69-95.

124. Martin R (1914) Lehrbuch der Anthropologie in systematischer Darstellung : mit besonderer Berücksichtigung der anthropologischen Methoden für Studierende Ärzte und Forschungsreisende. Stuttgart: Jena Verl. von Gustav Fischer. 1168 p.

125. Quam R, Bailey S, Wood B (2009) Evolution of M^1^ crown size and cusp proportions in the genus *Homo*. J Anat 214: 655-670.

126. Tafforeau P (2004) Aspects phylogénétiques et fonctionnels de la microstructure de l'émail dentaire et de la structure tri-dimentionnelle des molaires chez les primates fossiles et actuels: apports de la microtomographie à rayonnement X synchrotron. PhD University of Montpellier II. 284 p.

127. Olejniczak AJ, Grine FE (2006) Assessment of the Accuracy of Dental Enamel Thickness Measurements Using Microfocal X-Ray Computed Tomography. Anat Rec 288: 263-275.

128. Olejniczak AJ, Tafforeau P, Smith TM, Temming H, Hublin JJ (2007) Technical Note: Compatibility of Microtomographic Imaging Systems for Dental Measurements. Am J Phys Anthropol 134: 130-134.

129. Martin L (1985) Significance of enamel thickness in hominoid evolution. Nature 314: 260-263.

130. Grine FE, Martin LB (1988) Enamel Thickness and development in *Australopithecus* and *Paranthropus*. In: Grine FE, editor. Evolutionary History of the "Robust" Australopithecines. New York: Aldine de Gruyter. pp. 3-42.

131. Olejniczak AJ, Smith TM, Skinner MM, Grine FE, Feeney RNM, et al. (2008) Three-dimensional molar enamel distribution and thickness in *Australopithecus* and *Paranthropus*. Biol Lett 4: 406-410.

132. Smith TM, Olejniczak AJ, Zermeno JP, Tafforeau P, Skinner MM, et al. (2012) Variation in enamel thickness within the genus *Homo*. J Hum Evol 62: 395-411.

133. Olejniczak AJ, Smith TM, Feeney RNM, Macchiarelli R, Mazurier A, et al. (2008) Dental tissue proportions and enamel thickness in Neandertal and modern human molars. J Hum Evol 55: 12-23.

134. Martin L (1983) The relationships of the later Miocene Hominoidea. PhD University College London. 450 p.

135. Kono RT (2004) Molar enamel thickness and distribution patterns in extant great apes and humans: new insights based on a 3-dimensional whole crown perspective. Anthropol Sci 112: 121-146.

136. Grine FE (2005) Enamel thickness of deciduous and permanent molars in modern *Homo sapiens*. Am J Phys Anthropol 126: 14-31.

137. Olejniczak AJ (2006) Micro-Computed Tomography of Primate Molars. PhD Stony Brook University. 242 p.

138. Skinner MM, Gunz P, Wood BA, Hublin JJ (2009) Discrimination of extant *Pan* species and subspecies using the enamel-dentine junction morphology of lower molars. Am J Phys Anthropol 140: 234-243.

139. Skinner MM, Gunz P, Wood BA, Hublin JJ (2008) Enamel-dentine junction (EDJ) morphology distinguishes the lower molars of *Australopithecus africanus* and *Paranthropus robustus*. J Hum Evol 55: 979-988.

140. Bookstein FL (1997) Morphometric Tools for Landmark Data. Geometry and Biology Cambridge: Cambridge University Press. 456 p.

141. Gunz P, Mitteroecker P, Bookstein FL (2005) Semilandmarks in Three Dimensions. In: Slice DE, editor. Modern Morphometrics in Physical Anthropology. New York: Kluwer Academic/Plenum Publishers. pp. 73-98.

142. Gunz P, Mitteroecker P, Neubauer S, Weber GW, Bookstein FL (2009) Principles for the virtual reconstruction of hominin crania. J Hum Evol 57: 48-62.

143. Gower JC (1975) Generalized procrustes analysis. Psychometrika 40: 33-51.

144. Rohlf FG, Slice D (1990) Extensions of the Procrustes Method for the Optimal Superimposition of Landmarks. Sysr Biol 39: 40-59.

145. Dryden IL, Mardia KV (1998) Statistical shape analysis. Chichester: John Wiley & Sons. 347 p.

1. The back page list from the notebook is identical to the one given on the back page of the previous letter under the mention "Report Tuesday night 25". [↑](#footnote-ref-2)
2. Up to the 400th sample, which marks the end of the 1950's excavation at Ishango (the 18/VII) all the samples containing human remains where detailed the same way as sample 18. Although two isolated teeth were found at Ishango and marked as belonging to the NFPr layer, neither are mentioned in de Heinzelin’s notebook. [↑](#footnote-ref-3)
